# Scaling Limits of Multichannel Spectral Routers for Snapshot Imaging

**DOI:** 10.1002/nap2.70360

**Published:** 2026-11-13

**Authors:** Junseo Han, Seunghyun Lee, Donghyun Kim, Haejun Chung

**Affiliations:** ^1^ Department of Artificial Intelligence Semiconductor Engineering Hanyang University Seoul South Korea; ^2^ Department of Electronic Engineering Hanyang University Seoul South Korea

**Keywords:** delay‐bandwidth bound, inverse design, snapshot spectral imaging, spectral router, topology optimization

## Abstract

Inverse‐designed spectral routers can enable compact snapshot spectral imaging by directing different wavelengths to designated detector sub‐pixels without mechanical scanning or absorptive filters. This study examines how routing performance scales as the number of spectral channels increases over a fixed visible bandwidth. The routing condition is first formulated as a channel‐count‐dependent minimum separation between output states, then evaluated against Tucker‐ and Miller‐type delay‐bandwidth capacities to estimate the finite thickness required for a prescribed worst‐channel efficiency. Adjoint‐based topology optimization and three‐dimensional finite‐difference time‐domain simulations then yield TiO_2_/SiO_2_ routers with 9, 16, 25, and 36 channels under matched material, bandwidth, thickness, and optimization conditions. The average routing efficiency increases with sub‐pixel size and approaches a plateau, whereas the maximum near‐plateau efficiency decreases monotonically from 97.0% for 9 channels to 82.3% for 36 channels. At a fixed channel count, compressing the wavelength spacing to 8 nm or rearranging the wavelength‐to‐sub‐pixel assignment changes the efficiency by less than one percentage point. Therefore, the observed efficiency penalty is governed mainly by the number of wavelength‐dependent routing constraints rather than by wavelength spacing or local wavelength arrangement. This result indicates a practical trade‐off between spectral sampling and optical throughput.

## Graphical abstract

Inverse‐designed spectral routers enable compact snapshot imaging with multiple wavelength channels. Under matched design conditions, increasing the channel count from 9 to 36 reduces the maximum average routing efficiency from 97.0% to 82.3%. Controlled simulations show that wavelength spacing and arrangement have much smaller effects. The results reveal a practical trade‐off between spectral sampling and optical throughput.

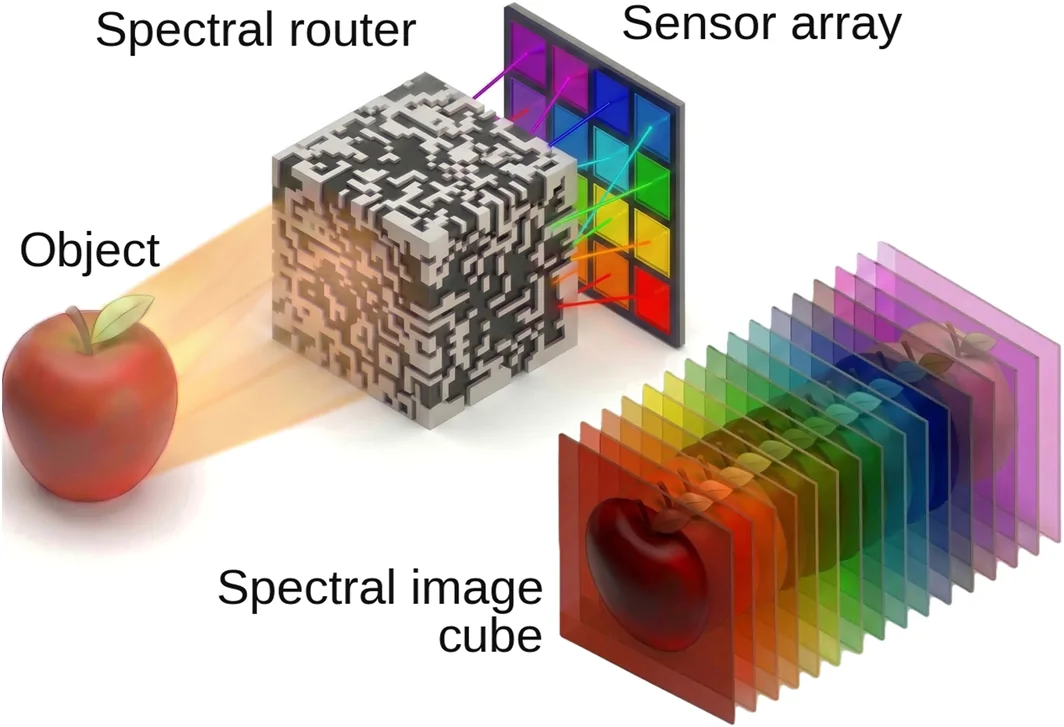

## 1 Introduction

Natural scenes are commonly modeled as radiance fields defined over continuous spatial and spectral coordinates, whereas practical image sensors reduce this continuum to a discrete array of spatially integrated and spectrally weighted measurements [1, 2, 3, 4, 5]. In the spatial dimension, a finite‐aperture imaging system imposes a diffraction‐ and aberration‐dependent optical transfer function with a finite optical bandwidth, and the resulting irradiance distribution is further integrated and sampled by finite‐area detector pixels [6, 7, 8, 9, 10]. In the spectral dimension, conventional single‐chip cameras map continuously varying scene radiance onto a small number of broad and overlapping detector responses, typically implemented using red, green, and blue color‐filter arrays, to reproduce color appearance rather than to recover wavelength‐resolved spectra [11, 12, 13, 14, 15]. As imaging systems become more compact and pixels continue to shrink, the benefit of finer spatial sampling is increasingly offset by reduced photon collection, diffraction‐limited blur, stronger angular dependence, and optical crosstalk between neighboring pixels [9, 14, 16, 17, 18, 19].

These trade‐offs are particularly important in mobile and pixel‐scale imagers, where the optical stack above each detector pixel must collect, spectrally select, and confine light within an increasingly small aperture [9, 17, 18]. As the pixel size approaches the wavelength scale, the microlens, color‐filter, and pixel‐isolation stack must simultaneously maintain high collection efficiency, preserve angular tolerance, and suppress optical crosstalk, whereas the achievable spatial resolution remains bounded by the diffraction‐limited passband of the optical front end [8, 9, 17, 18, 20, 21]. At the same time, absorptive color‐filter arrays reject or attenuate a substantial fraction of out‐of‐band photons, reducing the detected photoelectron count and thereby degrading the shot‐noise‐limited signal‐to‐noise ratio [9, 12, 14, 22]. These limitations have motivated sensor‐integrated wavelength‐sorting optics that redirect spectral components to designated detector regions rather than absorbing or rejecting them. Examples include color splitters, color routers, and metasurface‐based wavelength sorters, which can improve photon utilization relative to conventional absorptive filters [14, 16, 19, 23, 24, 25, 26, 27, 28, 29, 30, 31, 32].

This photon‐budget constraint becomes particularly acute when compact imagers are extended beyond RGB acquisition toward multispectral and hyperspectral sensing [33, 34, 35]. Many applications require wavelength‐resolved measurements, including material identification, biomedical sensing, precision agriculture, food inspection, and environmental monitoring [36, 37, 38, 39, 40, 41]. Conventional spectral imagers have enabled such capabilities using spatial or spectral scanning, dispersive optics, tunable filters, and filter‐array mosaics combined with computational reconstruction [33, 34, 42, 43, 44]. However, these architectures generally involve trade‐offs among optical throughput, acquisition speed, system volume, spatial sampling, and reconstruction fidelity [33, 34, 35, 45, 46, 47]. Recent compact and integrated spectral imagers have pursued this balance through miniaturized optics and on‐chip computational sensing [48, 49, 50]. For compact snapshot architectures, increasing the number of spectral channels within a fixed pixel size further tightens the trade‐off among photon utilization, spatial sampling, and spectral discrimination [30, 51, 52, 53, 54].

For detector‐integrated snapshot spectral imagers, spectral routers offer an alternative approach to addressing this trade‐off [30, 54, 55, 56]. Rather than attenuating out‐of‐band photons, a spectral router uses a shared nanophotonic structure to direct different wavelength components toward designated detector sub‐pixels (Figure 1a) [14, 29, 30]. When implemented at the super‐pixel level, such a device can perform wavelength separation within a compact device size and can, in principle, improve photon utilization relative to absorptive filter arrays [14, 19, 29, 30]. Adjoint‐based topology optimization designs a dielectric structure within a prescribed volume using gradient‐based full‐wave simulations [16, 23, 24, 57, 58, 59, 60]. Previous studies have demonstrated that filterless color splitters, color routers, and related diffractive architectures can enhance photon utilization [14, 19, 25, 26, 27, 28, 29, 30] and can extend wavelength‐selective routing beyond RGB operation toward snapshot multispectral imaging [30, 54]. Yet a basic question remains open: how does routing efficiency scale as the number of simultaneously addressed spectral channels increases over a fixed bandwidth?

**FIGURE 1:**
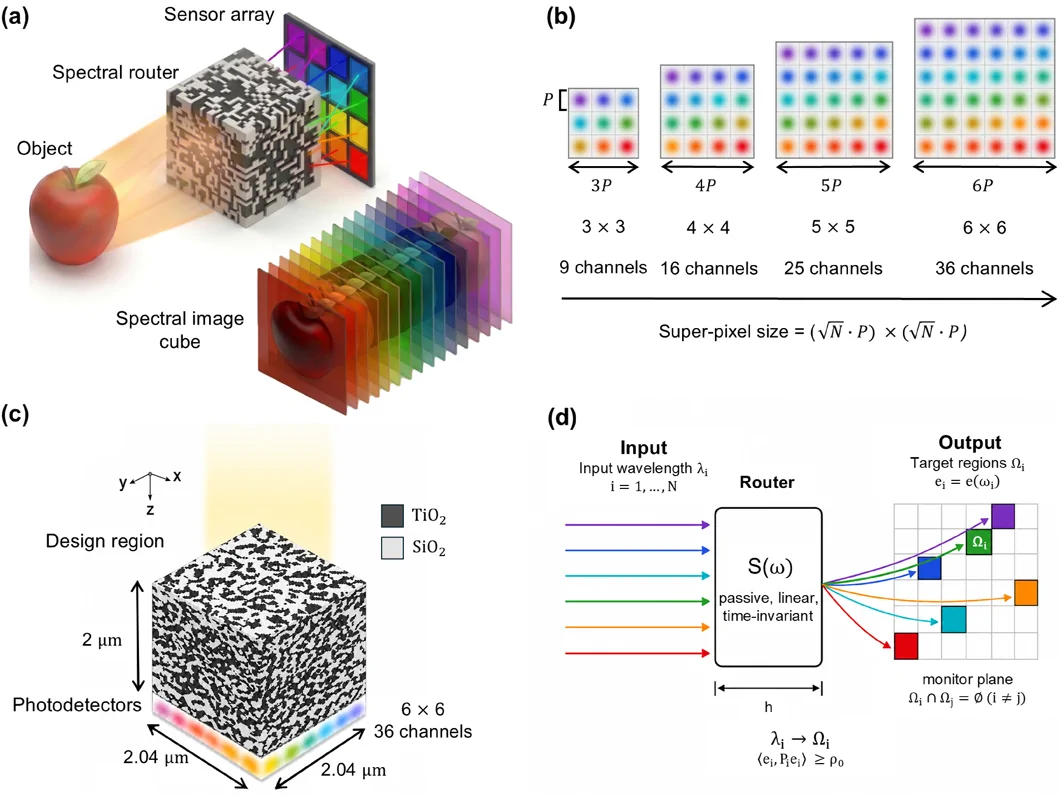
Concept and configuration of the inverse‐designed spectral router for snapshot spectral imaging. (a) Broadband light from an object is directed to wavelength‐dependent positions on the sensor array, enabling single‐shot acquisition of a spectral image cube. The conceptual illustration was created using Google Gemini 3 Pro Image (accessed March 2026) to generate an initial draft and subsequently refined and annotated by the authors. (b) Square super‐pixel configurations with N=9, 16, 25, and 36 channels, arranged as 3×3, 4×4, 5×5, and 6×6 sub‐pixel arrays, respectively. Each sub‐pixel has side length P, giving a super‐pixel size of $(\sqrt{N}P)\times \left(\sqrt{N}P\right)$. (c) Representative binary TiO_2_/SiO_2_ structure for the 36‐channel router, with lateral dimensions of 2.04μm×2.04μm and a design thickness of h=2μm, positioned above the photodetector plane. (d) Scattering‐operator description of the routing process. Each input wavelength $\lambda_{i}$ is mapped to a distinct target region $\Omega_{i}$ on the output monitor plane, where the incident‐power‐normalized output state $e_{i}=e\left(\omega_{i}\right)$ satisfies $\langle e_{i},P_{i}e_{i}\rangle \geq \rho_{0}$. The target regions are mutually nonoverlapping, $\Omega_{i}\cap \Omega_{j}=\emptyset$ for i≠j.

Answering this question requires disentangling several effects that vary together in a direct channel‐count sweep. Increasing the number of channels over a fixed spectral band simultaneously increases the number of routing constraints, reduces the spacing between adjacent target wavelengths, and modifies the local wavelength arrangement across neighboring sub‐pixels. This raises four questions. First, can a delay‐bandwidth bound perspective explain the efficiency degradation observed with increasing channel count? Second, how does the minimum device thickness required to maintain a prescribed worst‐channel efficiency scale with the number of channels, and conversely, what worst‐channel efficiency can a finite‐thickness structure support as more channels are added? Third, how do the optimized routing efficiencies of inverse‐designed routers depend on the channel count and the sub‐pixel size? Fourth, is the degradation driven mainly by the number of simultaneously routed channels or by the accompanying changes in wavelength spacing and local wavelength arrangement?

In this work, we address these questions by combining a delay‐bandwidth analysis with a systematic computational study of inverse‐designed spectral routers supporting 9, 16, 25, and 36 channels across the visible band. The bound analysis translates the routing condition into a minimum required delay‐bandwidth capacity that grows with both the number of channels and the prescribed worst‐channel efficiency. This capacity requirement is then expressed as a minimum device‐thickness estimate. Adjoint‐based topology optimization and finite‐difference time‐domain (FDTD) simulations then quantify the routing efficiencies obtained at a fixed device thickness and provide a numerical comparison with the trend indicated by these estimates. Targeted control studies based on compressed wavelength spacing and rearranged wavelength layouts further show that neither the reduced wavelength spacing nor the local wavelength arrangement accounts for the observed degradation, which instead tracks the number of simultaneously routed channels. These results delineate a practical scaling limit for spectral‐router‐based snapshot imaging.

## 2 Problem Formulation and Delay‐Bandwidth Bound

To analyze wavelength routing in terms of delay‐bandwidth capacity, we consider a finite‐thickness spectral router based on a dielectric metasurface, in which a normally incident plane wave at each discrete wavelength channel is directed to a prescribed, spatially separated output region. The metasurface is modeled as a passive, linear, and time‐invariant dielectric structure over the wavelength range of interest. The central question is how many spectrally distinct output states such a finite‐thickness structure can support within a given bandwidth while maintaining a prescribed routing efficiency. To answer this question, we derive the minimum state‐space separation imposed by the routing condition and compare the resulting trajectory‐length requirement with delay‐bandwidth limits for finite optical structures.

We define the routed spectrum in terms of N discrete wavelength channels $\lambda_{i}$ (i=1,…,N), distributed over the spectral interval $\left[\lambda_{\min},\lambda_{\max}\right]$. The corresponding angular frequencies are given by $\omega_{i}=2\pi c_{0}/\lambda_{i}$, where $c_{0}$ is the speed of light in vacuum. The incident‐power‐normalized output field state at $\omega_{i}$ on the monitor plane is denoted by $e_{i}=e\left(\omega_{i}\right)$, with $\|e_{i}{\|}^{2}=T_{\text{mon}}\left(\lambda_{i}\right)\leq 1$, where $T_{\text{mon}}=P_{\text{mon}}/P_{\text{inc}}$ is the ratio of total monitored output power to incident power. The target monitor regions $\Omega_{i}$ are mutually nonoverlapping, satisfying $\Omega_{i}\cap \Omega_{j}=\emptyset$ for i≠j. In the analytical output‐space model, $P_{i}$ is the orthogonal projection operator onto $\Omega_{i}$, with $P_{i}P_{j}=0$ for i≠j and 〈⋅,⋅〉 denotes the positive‐definite power inner product over the output field space. Since $e_{i}$ is normalized to the incident power, $\langle e_{i},P_{i}e_{i}\rangle$ represents the fraction of the incident power delivered to the corresponding target region $\Omega_{i}$. Therefore, the routing condition is written as

(1) $$\langle e_{i},P_{i}e_{i}\rangle \geq \rho_{0},\;i=1,\text{…},N,$$

where $\rho_{0}$ denotes the prescribed worst‐channel incident‐to‐target routing efficiency threshold.

This routing condition imposes a minimum separation between output states assigned to different target regions. For i≠j, the state $e_{j}$ contains at least a fraction $\rho_{0}$ of the incident power in $\Omega_{j}$, leaving at most a fraction $1-\rho_{0}$ in $\Omega_{i}$ because $\|e_{j}{\|}^{2}\leq 1$. Applying the reverse triangle inequality separately to the components projected by $P_{i}$ and $P_{j}$ gives $\|P_{k}\left(e_{i}-e^{i\theta }e_{j}\right)\|\geq \sqrt{\rho_{0}}-\sqrt{1-\rho_{0}}$ for k=i,j and any θ, provided $\rho_{0}>1/2$. Orthogonality then gives $\|e_{i}-e^{i\theta }e_{j}{\|}^{2}\geq 2{\left(\sqrt{\rho_{0}}-\sqrt{1-\rho_{0}}\right)}^{2}$. This gives the following bound on the phase‐invariant distance between the two output states:

(2) $$d\left(e_{i},e_{j}\right)\equiv \min_{\theta \in [0,2\pi )}\|e_{i}-e^{i\theta }e_{j}\|\geq \delta \left(\rho_{0}\right)=\sqrt{2-4\sqrt{\rho_{0}\left(1-\rho_{0}\right)}},$$

where a nonzero separation, $\delta \left(\rho_{0}\right)>0$, requires $\rho_{0}>1/2$. Otherwise, the same spatial pattern could in principle satisfy the routing condition for multiple disjoint target regions.

Although the routing condition is imposed only at discrete wavelength channels, the normalized output field state e(ω) varies continuously with angular frequency ω. As ω is swept across the band, the output state traces a continuous trajectory that passes through the N routed output states. The total length of this state‐space trajectory is quantified by $L_{\text{actual}}$, defined as the integral of the phase‐removed spectral derivative $\left\|D_{\omega }e\right\|$. Here, $D_{\omega }e$ denotes the phase‐removed derivative, with $\left\|D_{\omega }e\|=\min_{\alpha \in R}\|de/d\omega -i\alpha e\right\|$. This removes only an arbitrary global phase variation, whereas changes in total monitored transmission remain included. Since the trajectory must connect N output states in spectral order, and each successive pair is separated by at least $\delta \left(\rho_{0}\right)$, its length is bounded below by the sum of the corresponding chord distances:

(3) $$L_{\text{actual}}=\int_{\omega_{N}}^{\omega_{1}}\|D_{\omega }e\|d\omega \geq \left(N-1\right)\delta \left(\rho_{0}\right).$$

To connect the required state‐space trajectory length to the finite device thickness, we describe the metasurface as a frequency‐dependent linear scattering system. The output state defined above can be written as $e\left(\omega \right)=R_{\text{mon}}S\left(\omega \right)u_{0}$ for the incident state $u_{0}$, where S(ω) is the full scattering operator, including all transmitted and reflected power‐carrying channels and $R_{\text{mon}}$ selects the monitored output. We use a fixed power‐normalized channel representation with frequency‐independent $u_{0}$ and $R_{\text{mon}}$, $\|u_{0}\|=1$, and $\|R_{\text{mon}}\|\leq 1$. For this step, the full scattering system is assumed lossless, so S is unitary. The spectral rate of change of this normalized output state can be bounded in terms of the Wigner–Smith time‐delay operator, $Q_{\text{WS}}=-iS^{†}dS/d\omega$, as $\left\|D_{\omega }e\|\leq \|Q_{\text{WS}}\right\|$ [61, 62]. Here, operator norms are induced by the power inner product.

A common phase does not affect the trajectory length, so $\left\|D_{\omega }e\|\leq \|Q_{\text{WS}}-\tau_{\text{ref}}I\right\|$ for any real reference delay $\tau_{\text{ref}}\left(\omega \right)$. For a chosen reference delay, let $\Delta T_{\max}$ be an upper bound on this relative‐delay norm over the operating band. For the thickness estimate, we represent the relative‐delay response by an effective longitudinal delay line of length h, to which the selected delay‐bandwidth bound is assumed to apply. Therefore, we bound the trajectory length achievable over the bandwidth as $L_{\text{actual}}\leq \Delta T_{\max}\Delta \omega \leq \kappa \left(h\right)$, where κ(h) denotes the dimensionless delay‐bandwidth capacity of a structure with thickness h [63] and $\Delta \omega =\omega_{1}-\omega_{N}$. Combining this upper bound with the lower‐bound requirement in Equation (3) yields the necessary condition within this delay‐line model:

(4) $$\left(N-1\right)\delta \left(\rho_{0}\right)\leq \kappa \left(h\right).$$

To translate this condition into a thickness estimate, we next specify the delay‐bandwidth capacity κ(h) for a finite‐thickness metasurface. Because κ(h) depends on the physical model used to describe delay accumulation in the structure, we consider two representative bounds. First, we use a Tucker‐type propagation‐delay estimate for a waveguide‐like nonresonant dielectric structure [64]:

(5) $$\kappa_{\text{Tucker}}\left(h\right)=2\pi \frac{h}{\lambda_{c}}\Delta n,$$

where $\lambda_{c}$ is the free‐space center wavelength and $\Delta n=n_{\max}-n_{\min}$ is the available refractive‐index contrast. This form of the bound is appropriate when the spectral response can be interpreted as propagation through transparent dielectric segments with a well‐defined group delay. Here, the constituent‐index contrast bounds the modal effective‐index range in the assumed waveguide model. The stated expression assumes a fractional bandwidth below unity. Substituting Equation (5) into Equation (4) yields the required thickness:

(6) $$h_{\min}^{\text{Tucker}}=\frac{\lambda_{c}}{2\pi \Delta n}\left(N-1\right)\delta \left(\rho_{0}\right).$$

As a second estimate, we adopt Miller's delay‐bandwidth bound, which applies to a broader class of one‐dimensional delay structures without requiring transparent materials or a well‐defined group velocity [65]. In this formulation, the delay‐bandwidth capacity is determined by the physical thickness of the structure and the relative permittivity contrast with respect to the background medium:

(7) $$\kappa_{\text{Miller}}\left(h\right)=\frac{\pi }{\sqrt{3}}\frac{h}{\lambda_{c,b}}\eta_{\max},$$

where $\lambda_{c,b}$ is the center wavelength measured in the background medium and $\eta_{\max}=|\varepsilon_{\max}-\varepsilon_{b}|/\varepsilon_{b}$ is the maximum relative permittivity contrast normalized by the background permittivity $\varepsilon_{b}$, following Miller's notation. The stated expression assumes a small fractional bandwidth and a device length large compared with $\lambda_{c,b}$. Applying the same necessary condition to Equation (7) gives the Miller‐bound thickness estimate:

(8) $$h_{\min}^{\text{Miller}}=\frac{\sqrt{3}\lambda_{c,b}}{\pi \eta_{\max}}\left(N-1\right)\delta \left(\rho_{0}\right).$$

These estimates do not determine the relative contributions of propagation and resonant energy storage in the optimized structure.

We evaluate these thickness bounds for the TiO_2_/SiO_2_ binary design region used in the optimized routers. Tucker's bound is evaluated using the TiO_2_/SiO_2_ refractive‐index contrast (Δn=1.20), whereas Miller's bound is evaluated by taking SiO_2_ as the background medium, giving $\eta_{\max}=2.3401$. For the 400–700 nm spectral range, the center wavelength is $\lambda_{c}=509.09\;nm$, corresponding to the wavelength at the midpoint of the frequency interval, and the background‐medium center wavelength used for Miller's bound is $\lambda_{c,b}=\lambda_{c,SiO_{2}}=351.10\;nm$. With these parameters, the resulting thickness estimates are summarized in Table 1.

**TABLE 1:** Delay‐bandwidth thickness estimates for a TiO_2_/SiO_2_ spectral router with N=36 channels spanning 400–700nm. The Tucker‐type estimate uses $\lambda_{c}=509.09\;nm$ and Δn=1.20, whereas the Miller‐type estimate uses SiO_2_ as the background medium, with $\lambda_{c,SiO_{2}}=351.10\;nm$ and $\eta_{\max}=2.3401$. The table reports $\delta \left(\rho_{0}\right)$ and the corresponding minimum‐thickness estimates for each prescribed threshold $\rho_{0}$.

$\rho_{0}$	$\delta \left(\rho_{0}\right)$	$h_{\min}^{\text{Tucker}}$	$h_{\min}^{\text{Miller}}$
0.8	0.6325	1.49μm	1.83μm
0.9	0.8944	2.11μm	2.59μm
1.0	1.4142	3.34μm	4.09μm

Figure 2 compares the simulated worst‐channel routing efficiencies with the Tucker‐type and Miller‐type delay‐bandwidth thickness estimates and visualizes their dependence on channel count and routing‐efficiency threshold. In Figure 2a, the solid and dashed curves denote the theoretical Tucker and Miller estimates, respectively, whereas the markers indicate the simulated inverse‐designed routers, plotted according to their achieved worst‐channel efficiency $\rho_{0,\min}$ and physical design thickness. Here, the achieved $\rho_{0,\min}$ of each simulated design is used as the numerical counterpart to the prescribed threshold $\rho_{0}$ in the state‐space bound. The theoretical curves show that the minimum thickness required to support a prescribed routing‐efficiency threshold increases with both the number of wavelength channels N and the target efficiency $\rho_{0}$. This behavior follows directly from the required state‐space trajectory length, $(N-1)\delta \left(\rho_{0}\right)$: increasing N introduces more routed output states that must be connected across the spectrum, whereas increasing $\rho_{0}$ requires stronger separation between states assigned to different target regions.

**FIGURE 2:**
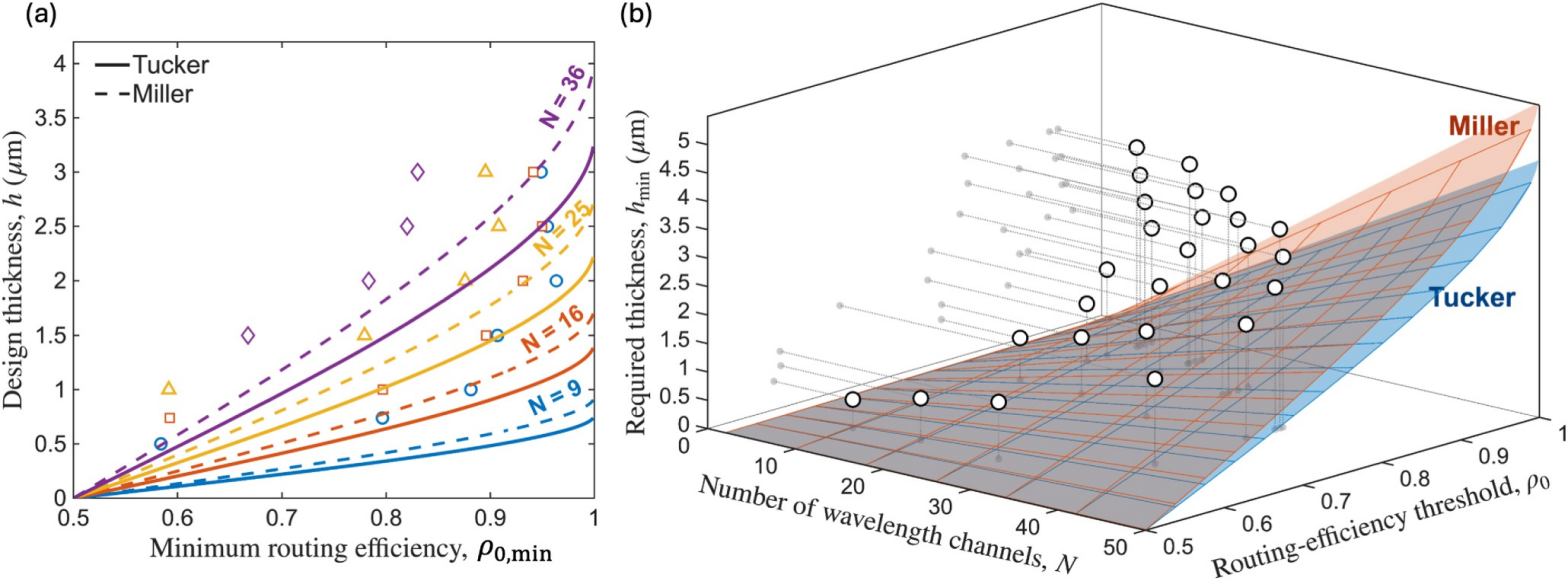
Comparison between simulated inverse‐designed routers and Tucker‐type [64] and Miller‐type [65] delay‐bandwidth thickness estimates. (a) Thickness estimates as functions of the routing‐efficiency threshold for different channel counts N. The markers denote the optimized spectral routers (this work) plotted using their achieved worst‐channel efficiency $\rho_{0,\min}$ and physical design thickness; at each thickness, the largest $\rho_{0,\min}$ over the simulated sub‐pixel sizes is shown (Supporting Information S1: Supplementary Sec. S9). (b) Three‐dimensional minimum‐thickness estimates as functions of channel count N and prescribed threshold $\rho_{0}$. The open markers indicate the optimized spectral router designs.

The simulated designs are broadly consistent with this tendency: structures with larger N generally require greater thickness to attain comparable worst‐channel efficiencies. However, the simulated efficiencies are not strictly monotonic with thickness. A larger design thickness increases the available optical degrees of freedom and can raise the attainable performance ceiling, but it does not guarantee that a particular optimization run will converge to a better design. Because the inverse‐design problem is highly nonconvex, different thicknesses can lead to different local optima, and the optimized efficiency may vary with initialization, parameterization, and finite iteration count. Therefore, nonmonotonic variations among the simulated markers should not be interpreted as violations of the delay‐bandwidth estimates. Rather, the bounds indicate the minimum capacity required to support a prescribed routing‐efficiency threshold, whereas the markers correspond to specific numerically optimized designs obtained under finite computational constraints.

Figure 2b extends this comparison to the full $\left(N,\rho_{0}\right)$ parameter space by plotting the Tucker‐type and Miller‐type estimates as three‐dimensional thickness‐bound surfaces. Both surfaces increase with the number of wavelength channels N and the prescribed routing‐efficiency threshold $\rho_{0}$, showing that routing more channels or enforcing a higher worst‐channel efficiency requires a larger delay‐bandwidth capacity and therefore a greater device thickness. The simulated inverse‐designed routers are overlaid using their achieved minimum efficiencies $\rho_{0,\min}$ and physical design thicknesses. These markers are not expected to lie exactly on the theoretical surfaces, because the surfaces represent necessary delay‐bandwidth capacity estimates rather than specific optimization outcomes. Nevertheless, the simulated results are consistent with the same overall tendency: routers with larger channel counts and higher achieved efficiencies lie in regions of larger thickness.

## 3 Numerical Results

This section presents the simulated routing performance of the optimized spectral routers. The delay‐bandwidth analysis provides a qualitative explanation for the increasing difficulty of routing more spectral channels, whereas the efficiency trends are determined directly from controlled adjoint‐based optimization and FDTD simulations. The numerical results are therefore interpreted independently of any quantitative fit to the theoretical estimates.

### 3.1 Router Configurations and Evaluation Metrics

We investigate inverse‐designed spectral routers that direct each incident wavelength to a designated detector sub‐pixel, enabling snapshot spectral imaging without mechanical scanning or absorptive filters. This approach builds on sensor‐integrated wavelength‐sorting devices, including color splitters and color routers, in which passive dielectric structures redistribute spectral components among detector regions rather than rejecting out‐of‐band light [14, 19, 29, 66, 67, 68]. Recent inverse‐designed color routers and freeform metasurfaces have demonstrated that such wavelength‐selective routing can be implemented within compact optical stacks compatible with image sensors [16, 23, 24]. Here, we examine higher‐channel‐count spectral routers and quantify how their attainable routing efficiency varies with the number of simultaneously routed wavelengths.

We consider square super‐pixel routers comprising N=k×k sub‐pixels, with N=9, 16, 25, and 36 corresponding to k=3, 4, 5, and 6, respectively. Each sub‐pixel is assigned a target wavelength within the visible spectrum, and the investigated sub‐pixel sizes cover submicrometer dimensions relevant to modern CMOS image sensors [9, 14]. As shown in Figure 1, the router consists of a finite‐thickness binary TiO_2_/SiO_2_ design region positioned above the detector plane. For each channel configuration, the dielectric distribution is optimized using adjoint‐based topology optimization and evaluated by three‐dimensional FDTD simulations [60, 69]. Further details are provided in Supporting Information S1: Supplementary Sec. S1. During optimization, we maximize a negative‐p‐norm objective (p=−4) of the channel routing efficiencies. This differentiable soft minimum emphasizes lower‐efficiency channels and encourages balanced routing across the target wavelengths. The average routing efficiency reported below is evaluated separately from this optimization objective.

We quantify the routing performance using the average routing efficiency,

$${\bar{\eta }}_{\text{route}}=\frac{1}{N}\sum_{i=1}^{N}T_{ii}\left(\lambda_{i}\right),$$

where $T_{ii}\left(\lambda_{i}\right)$ is the fraction of incident power at wavelength $\lambda_{i}$ delivered to its designated sub‐pixel i. With $T_{ii}$ normalized to incident power, both reflected power and power transmitted outside the target sub‐pixel reduce the reported routing efficiency. Total through‐transmission $T_{\text{tot}}\left(\lambda \right)$ instead measures all forward power crossing the full output plane divided by incident power, including power transmitted to nontarget regions. Supporting Information S1: Supplementary Sec. S10 compares $T_{ii}$ with the fraction of transmitted power delivered to each target region. The delay‐bandwidth analysis uses a prescribed worst‐channel efficiency threshold $\rho_{0}$. For comparison with this criterion, the simulated worst‐channel efficiency is defined as

$$\rho_{0,\min}=\min_{i}T_{ii}\left(\lambda_{i}\right).$$

For the channel‐count scaling study, the sub‐pixel size P is varied from 0.12μm to 0.60μm. Unless stated otherwise, all routers have a thickness of h=2μm and are simulated at an FDTD resolution of 50pixels/μm. The wavelength‐spacing and wavelength‐arrangement control studies are performed at P=0.34μm, allowing direct comparison at a fixed sub‐pixel size. For visualization, the focal‐plane $S_{z}$ distributions are cropped around their target sub‐pixels and assembled into composite panels. Each map is normalized independently to its peak value solely for display. All routing efficiencies are calculated from the simulated power flux normalized to the incident power and are unaffected by this visualization procedure.

### 3.2 Efficiency Scaling With Sub‐Pixel Size and Channel Count

We evaluate the average routing efficiency for N=9, 16, 25, and 36 spectral channels as the sub‐pixel size P varies from 0.12μm to 0.60μm. The resulting dependence on P and N is shown in Figure 3d.

**FIGURE 3:**
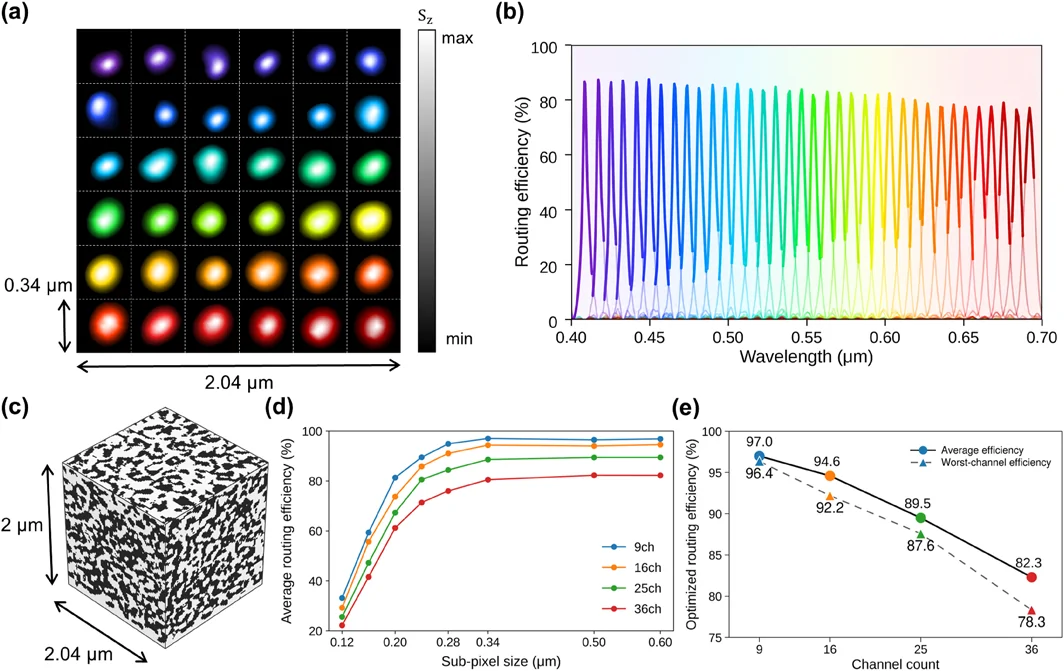
Channel‐count‐dependent scaling of routing efficiency. (a) Composite focal‐plane Poynting vector $S_{z}$ distributions for the optimized 36‐channel router at P=0.34μm, corresponding to a super‐pixel size of 2.04μm×2.04μm. Each channel map is cropped around its target sub‐pixel and normalized independently to its peak value for visualization. (b) Routing‐efficiency spectra of the 36‐channel router across the visible band. Each curve corresponds to one target wavelength and its designated detector region. (c) Optimized TiO_2_/SiO_2_ structure of the 36‐channel router at P=0.34μm, with a lateral size of 2.04μm×2.04μm and a thickness of 2μm. Light is normally incident on the top surface. (d) Average routing efficiency as a function of sub‐pixel size P over 0.12μmto0.60μm for *N* = 9, 16, 25, and 36 channels. For all channel counts, the efficiency increases with P and approaches a plateau for P≥0.34μm. (e) Average (solid circles) and worst‐channel (dashed triangles) routing efficiencies versus channel count at h=2μm. Both values for each N are evaluated for the same design selected from the sampled near‐plateau region.

Snapshot spectral imaging beyond RGB has been demonstrated using several meta‐optical approaches, including metasurface‐enabled snapshot hyperspectral imaging [70], six‐channel spectral routers [30], diffractive optical networks for 4, 9, and 16 spectral bands [54], and an eight‐channel chromatic metalens array imager spanning the visible and near‐infrared range [71]. These studies establish high‐channel‐count operation but do not isolate the effect of channel count under matched design conditions. Here, the 9‐, 16‐, 25‐, and 36‐channel routers are optimized and evaluated using the same materials, device thickness, spectral bandwidth, and FDTD‐based inverse‐design procedure.

For all channel counts, the average routing efficiency increases with P and approaches a plateau for P≥0.34μm as the larger lateral design area provides additional optical degrees of freedom. Two competing factors govern this behavior. Enlarging P increases both the lateral extent of the design region and the separation between neighboring target sub‐pixels, which relaxes the spatial confinement required of each routed field. At the same time, the target regions must remain distinguishable on the scale of the operating wavelengths. When P falls well below this scale, diffraction prevents the routed power from being localized within a single target sub‐pixel, and the efficiency drops rapidly. Beyond the plateau onset, further enlargement yields only modest gains, indicating that the lateral dimension is no longer the limiting resource. Figure 3a,b shows the focal‐plane $S_{z}$ distributions and routing‐efficiency spectra of the 36‐channel router at P=0.34μm.

Within the near‐plateau region, both the average and worst‐channel routing efficiencies decrease with channel count as shown in Figure 3e. For each N, we select the design with the highest observed average efficiency among the sampled P≥0.34μm and evaluate both metrics for that same design. The selected P values are 0.34μm, 0.60μm, 0.60μm, and 0.50μm for N=9, 16, 25, and 36, respectively. The corresponding average efficiencies are 97.0%, 94.6%, 89.5%, and 82.3%, whereas the worst‐channel efficiencies are 96.4%, 92.2%, 87.6%, and 78.3%. Under the matched design conditions considered here, this decline indicates that directing more wavelengths to distinct locations within a compact super‐pixel imposes increasingly demanding routing constraints.

The achieved worst‐channel efficiencies, $\rho_{0,\min}$, are compared with the delay‐bandwidth estimates in Section 2 and Figure 2. For the fixed thickness h=2μm, the Miller estimate is more restrictive than the Tucker estimate for the TiO_2_/SiO_2_ system. It permits unity worst‐channel efficiency for N=9 and 16, but limits the corresponding values to approximately 0.94 and 0.82 for N=25 and 36, respectively. The simulated $\rho_{0,\min}$ values remain below these estimates, with the higher‐channel‐count routers operating closer to the predicted capacity limit. This trend is consistent with the interpretation that increasing N raises the delay‐bandwidth demand of a finite‐thickness router. However, the estimate provides a necessary capacity condition rather than an exact performance bound for the three‐dimensional optimized structures.

The following sections assess whether the observed efficiency degradation arises from reduced wavelength spacing, wavelength arrangement, or the increased number of simultaneously routed channels.

### 3.3 Channel Count Versus Wavelength Spacing

The channel‐count sweep changes both the number of routing targets and the spacing between adjacent wavelengths. Across the 400–700 nm band, the baseline 9‐, 16‐, 25‐, and 36‐channel routers use nominal spacings of Δλ=30nm, 20nm, 12nm, and 8nm, respectively. Integer‐nanometer wavelength sampling introduces slight nonuniformity, with 15nm and 20nm steps in the 16‐channel set and 8nm and 9nm steps in the 36‐channel set. Therefore, the efficiency decline observed as N increases may arise from either the greater number of routing constraints or the narrower wavelength spacing.

A dependence on wavelength spacing is physically plausible because resolving closely spaced spectral channels generally requires a longer effective optical path or a narrower spectral response [72, 73]. Dense spectral sampling is also relevant to snapshot spectral imaging. For example, a parallel metasystem resolves 20 channels over 795nmto980nm, corresponding to an average spacing of approximately 10nm [74]. However, prior demonstrations vary channel spacing together with other device parameters and therefore do not isolate its effect at a fixed channel count.

To isolate the effect of wavelength spacing, we introduce the compressed‐spacing controls shown in Figure 4d–f, with the corresponding full‐super‐pixel $S_{z}$ maps provided in Supporting Information S1: Supplementary Sec. S8. For each lower‐channel‐count router, the channel count and spatial target layout are unchanged, whereas the target wavelengths are reassigned with a nominal spacing of 8nm, matching the 36‐channel case and centered within the visible band. This comparison separates the influence of wavelength spacing from that of channel count. All compressed‐spacing designs are evaluated at P=0.34μm, where the routing efficiency is near its plateau.

**FIGURE 4:**
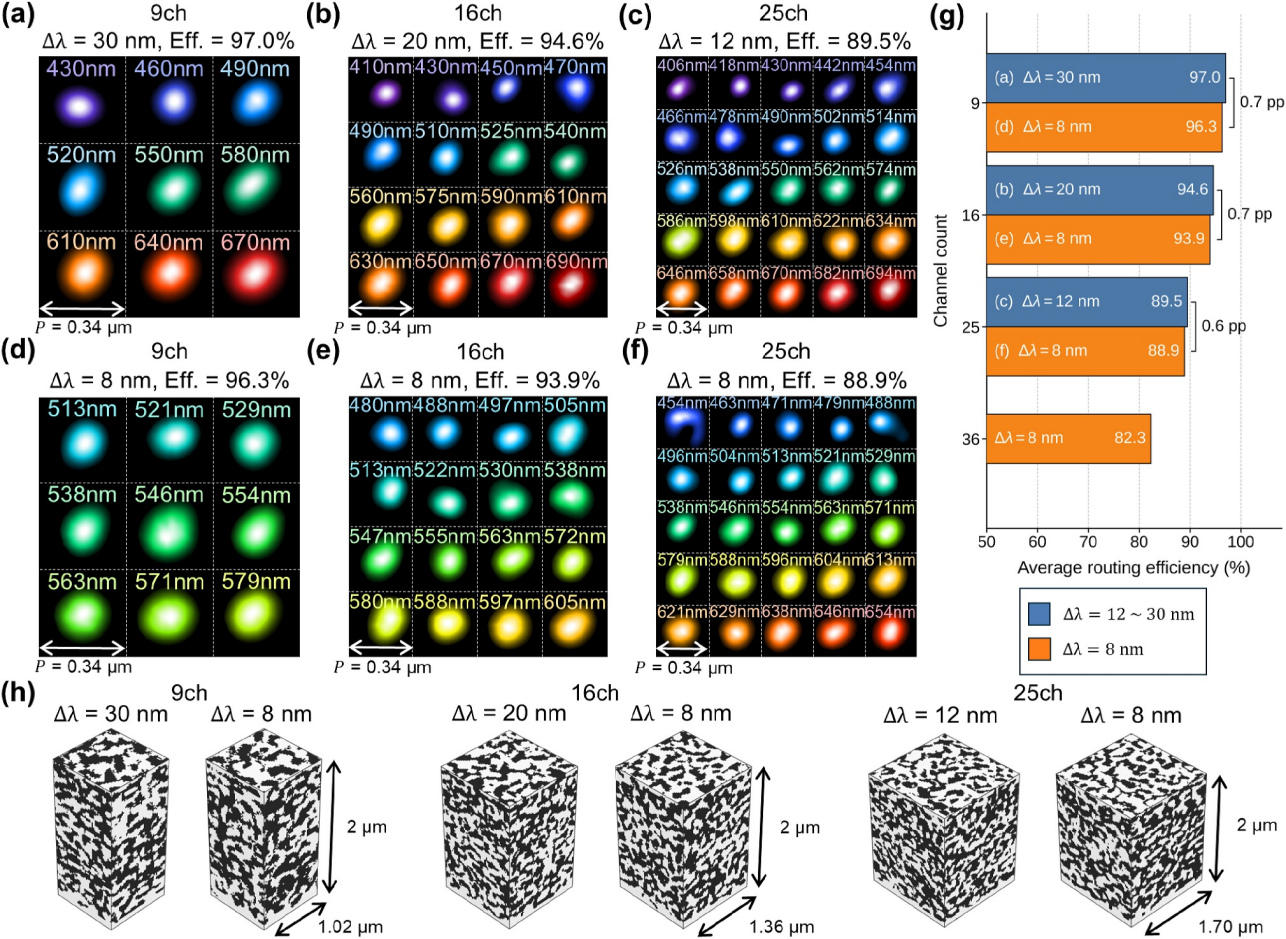
Effect of wavelength spacing on routing efficiency. (a)–(c) Composite focal‐plane $S_{z}$ distributions for the 9‐, 16‐, and 25‐channel routers using their channel‐count‐dependent wavelength spacings of Δλ=30nm, 20nm, and 12nm, respectively. (d)–(f) Corresponding compressed‐spacing designs with the same channel counts and spatial target layouts but a nominal spacing of Δλ=8nm. Each channel map is cropped around its target sub‐pixel and normalized independently to its peak value for visualization. (g) Comparison of the average routing efficiencies for the channel‐count‐dependent and compressed wavelength spacings. Reducing Δλ to 8nm lowers the efficiency by only 0.6–0.7 percentage points for the 9‐, 16‐, and 25‐channel routers, substantially less than the decrease observed with increasing channel count. The 36‐channel result is included as the Δλ=8nm reference. (h) Corresponding optimized TiO_2_/SiO_2_ structures for the six configurations shown in (a)–(f), all evaluated at P=0.34μm with a thickness of 2μm. Dark and light regions denote TiO_2_ and SiO_2_, respectively.

Compressing the wavelength spacing produces only a minor reduction in average routing efficiency. The matched‐spacing references are the maximum near‐plateau efficiencies reported in Figure 3e, making this a conservative comparison with the compressed‐spacing designs evaluated at P=0.34μm. The efficiency decreases from 97.0% to 96.3% for N=9, from 94.6% to 93.9% for N=16, and from 89.5% to 88.9% for N=25, corresponding to reductions of 0.7, 0.7, and 0.6 percentage points, respectively. These changes are much smaller than the 14.7‐percentage‐point difference between the 9‐ and 36‐channel routers. Thus, reducing the wavelength spacing to 8nm at fixed N does not account for the efficiency degradation observed with increasing channel count.

This result does not imply that wavelength spacing is unimportant in general. However, within the spectral range and device conditions considered here, compressing the spacing to 8nm at fixed N produces a much smaller efficiency change than increasing the number of routed channels. The observed degradation therefore cannot be attributed to wavelength spacing alone.

### 3.4 Channel Count Versus Wavelength Arrangement

We next examine whether the spatial assignment of wavelengths affects routing efficiency. In the ${\bar{\Delta \lambda }}_{\text{adj}}$‐minimized arrangement, channels are assigned sequentially in wavelength, placing spectrally similar channels in neighboring sub‐pixels. Such proximity could, in principle, increase susceptibility to local crosstalk between adjacent detector regions.

The influence of output‐channel arrangement has also been examined in multichannel color routers. A recent diffractive‐neural‐network study tested ten randomly selected output configurations for a 9‐channel router and reported little variation in average crosstalk or peak wavelength response [75]. Here, we instead construct a deterministic arrangement that maximizes the mean spectral separation between adjacent sub‐pixels and compare it directly with the sequential wavelength assignment.

To evaluate the effect of wavelength arrangement, we compare the ${\bar{\Delta \lambda }}_{\text{adj}}$‐minimized configuration of the 36‐channel router with a configuration that maximizes the mean spectral separation between adjacent sub‐pixels among the modular arrangements described in Supporting Information S1: Supplementary Sec. S3. As shown in Figure 5a,e, ${\bar{\Delta \lambda }}_{\text{adj}}$ increases from 48.67nm to 116.67nm. The corresponding focal‐plane $S_{z}$ distributions and per‐channel routing efficiencies are presented in Figure 5b,f and Figure 5c,g, respectively.

**FIGURE 5:**
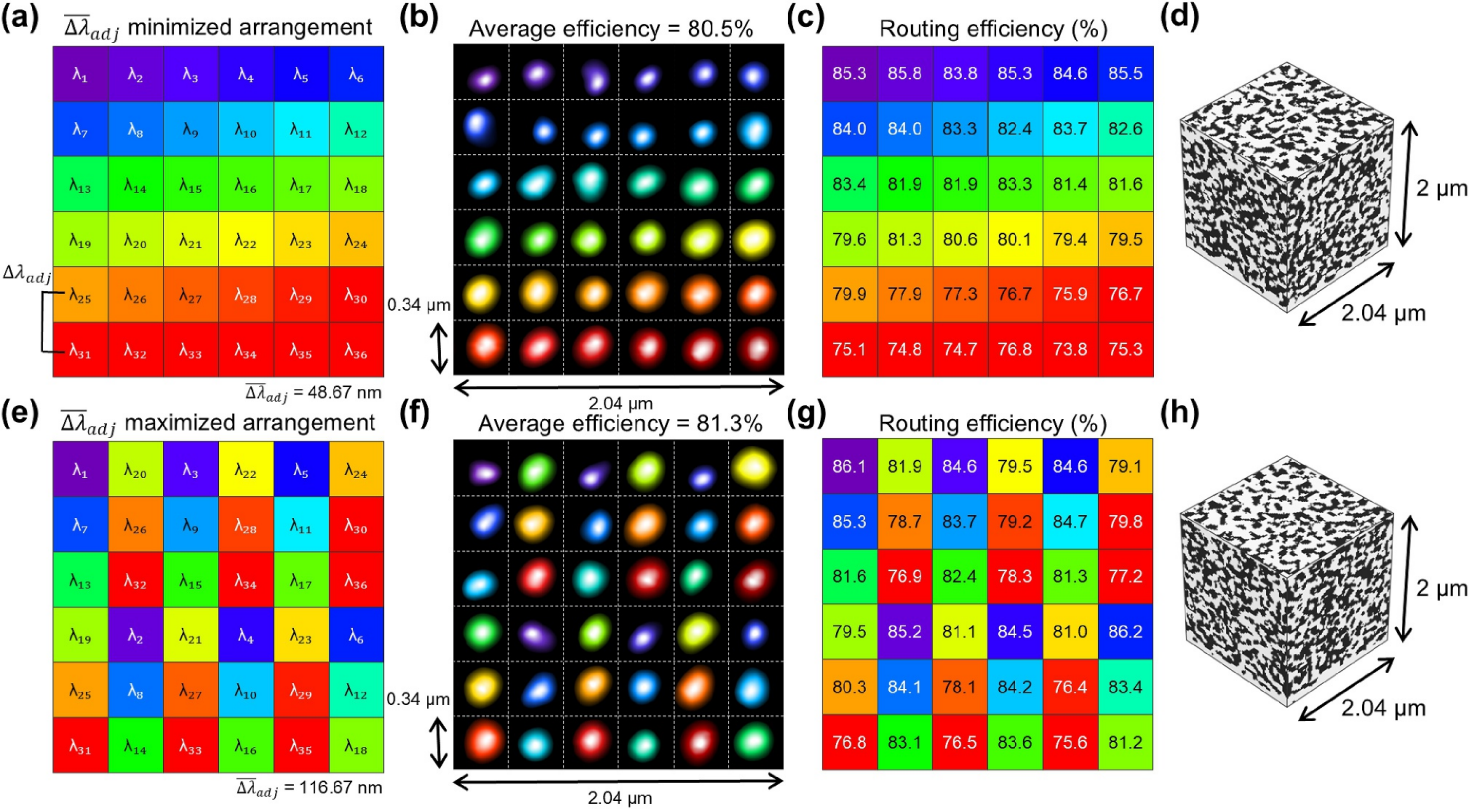
Limited effect of wavelength arrangement on routing efficiency. Two 36‐channel arrangements are compared at P=0.34μm: the ${\bar{\Delta \lambda }}_{\text{adj}}$‐minimized arrangement [(a)–(d)] and the ${\bar{\Delta \lambda }}_{\text{adj}}$‐maximized arrangement [(e)–(h)], where ${\bar{\Delta \lambda }}_{\text{adj}}$ denotes the average wavelength difference between spatially adjacent sub‐pixels. (a), (e) Wavelength‐to‐sub‐pixel assignment maps, with ${\bar{\Delta \lambda }}_{\text{adj}}=48.67\;nm$ in panel (a) and 116.67nm in panel (e). (b), (f) Corresponding composite focal‐plane $S_{z}$ distributions, with average routing efficiencies of 80.5% and 81.3%, respectively. Each channel map is cropped around its target sub‐pixel and normalized independently to its peak value for visualization. (c), (g) Per‐channel routing‐efficiency maps arranged according to the sub‐pixel positions in panels (a) and (e). (d), (h) Corresponding optimized TiO_2_/SiO_2_ structures with a thickness of 2μm. The average routing efficiency changes by only 0.8 percentage points, indicating that wavelength arrangement has only a minor effect compared with the channel‐count‐dependent degradation observed in Figure 3.

All wavelength‐arrangement comparisons are evaluated at P=0.34μm. At this sub‐pixel size, the sequential configuration yields an average routing efficiency of 80.5%, whereas the 82.3% value reported in Figure 3 denotes the maximum efficiency over the near‐plateau range P≥0.34μm.

Despite the larger spectral separation between neighboring sub‐pixels, the average routing efficiency changes only from 80.5% to 81.3%, an increase of 0.8 percentage points. Thus, under the conditions tested, wavelength arrangement has only a minor influence on routing efficiency. This variation is substantially smaller than the efficiency reduction caused by increasing the number of simultaneously routed channels.

These results indicate that local wavelength adjacency is not the dominant limitation for the 36‐channel router under the tested conditions. The larger performance penalty is instead associated with requiring a finite‐thickness structure to map many spectrally distinct inputs to separate nonoverlapping detector regions.

## 4 Discussion

Among the variables examined at a fixed sub‐pixel size, channel count had the strongest effect on routing efficiency. Compressing the wavelength spacing to Δλ=8nm reduced the average efficiency by only 0.6–0.7 percentage points for the 9‐, 16‐, and 25‐channel routers, whereas rearranging the 36 target wavelengths to increase the spectral separation between adjacent sub‐pixels changed the efficiency by less than one percentage point. These variations are much smaller than the 14.7‐percentage‐point decrease observed between the 9‐ and 36‐channel designs. Therefore, under the material, thickness, bandwidth, and optimization conditions considered here, the efficiency degradation cannot be attributed primarily to narrower wavelength spacing or local wavelength arrangement. Instead, it is associated mainly with the increasing number of wavelength‐dependent field distributions that must be generated and directed to nonoverlapping detector regions by a compact, finite‐thickness dielectric structure.

This interpretation is consistent with the delay‐bandwidth analysis. As the number of channels increases within a fixed spectral band, the router must generate more mutually distinct output states, increasing the required state‐space trajectory length across frequency. A finite‐thickness structure therefore needs greater delay‐bandwidth capacity to maintain a given worst‐channel efficiency. The observed decline in near‐plateau efficiency is consistent with this increasing demand at fixed thickness. However, the bound should be interpreted as a necessary capacity estimate that explains the trend rather than as a quantitative prediction of the optimized efficiencies. The optimized router is a volumetric dielectric scatterer that redistributes power laterally through wavelength‐dependent interference. At h=2μm, its thickness spans approximately 2.9–5.0 free‐space wavelengths over 400–700 nm, allowing propagation phase to accumulate. However, the simulated output‐field maps and routing spectra do not separate propagation delay from resonant energy storage. Therefore, we do not infer that either contribution dominates and use the Tucker‐ and Miller‐type expressions as model‐dependent thickness estimates. Although the Tucker‐ and Miller‐type delay‐bandwidth capacities increase linearly with h under their respective assumptions, the thickness sweep in Supporting Information S1: Supplementary Sec. S9 shows diminishing gains or nonmonotonic changes in average routing efficiency for some configurations beyond approximately h=2μm. These trends are consistent with possible limitations of finite, local optimization in a nonconvex design space, but the sweep alone cannot determine their contribution.

These results have direct implications for compact snapshot spectral imagers. Increasing the channel count improves spectral sampling but reduces routing efficiency in compact super‐pixel geometries. Enlarging the sub‐pixel improves efficiency only until the response approaches a plateau for P≥0.34μm. Therefore, further lateral scaling provides diminishing returns. Practical designs should balance channel count, sub‐pixel size, device thickness, and spectral bandwidth according to the required spectral resolution and available photon budget.

Several limitations should be considered. The routers considered in the main channel‐scaling analysis were evaluated using idealized FDTD models that exclude fabrication errors, material variations, detector noise, and image‐level spectral reconstruction. Supporting Information S1: Supplementary Sec. S14 examines the effect of an imposed minimum feature size and shows that a 60 nm constraint preserves near‐baseline simulated routing efficiency. Lateral TiO_2_ features at this scale can be fabricated, but achieving suitable etch profiles and layer alignment throughout the proposed multilayer structure in a CMOS‐compatible process remains to be demonstrated. Supporting Information S1: Supplementary Sec. S15 shows that uniform erosion‐ and dilation‐like perturbations at the TiO_2_/SiO_2_ interfaces shift the channel peak wavelengths of a representative 36‐channel router. As these analyses address only selected fabrication effects, a full fabrication‐tolerance analysis remains necessary. Topology optimization may converge to local optima. The reported efficiencies were obtained with identical optimization settings for all designs, with additional random initializations for selected designs, and therefore do not represent global limits. The main channel‐scaling results were obtained at normal incidence with 45° linearly polarized illumination, primarily at a fixed thickness of h=2μm. Thickness dependence is examined separately in Supporting Information S1: Supplementary Sec. S9. Polarization‐independent and wide‐angle performance therefore remains to be demonstrated. Including both orthogonal polarizations in the optimization objective can reduce polarization dependence as shown for a representative 36‐channel design in Supporting Information S1: Supplementary Sec. S11. Polarization‐resolved behavior across all channel counts remains to be characterized. Operation over the range of incidence angles encountered in a practical imaging system remains beyond the scope of the present normal‐incidence designs. Angle‐specific routers assigned to different regions of the sensor, with structural‐similarity constraints at their boundaries, offer a possible approach to this angular limitation [76]. Whether this approach extends to multichannel spectral routing at the channel counts studied here remains to be investigated. Supporting Information S1: Supplementary Sec. S13 provides an initial extension to the visible–near‐infrared range, whereas systematic studies of other spectral bands remain open.

Future studies should incorporate fabrication‐aware optimization, experimental validation, and image‐level reconstruction. Greater device thickness or designs with additional optical degrees of freedom may improve routing performance at high channel counts, whereas joint optimization of the router and reconstruction algorithm [77, 78, 79] could better balance optical throughput, spectral discrimination, and reconstruction fidelity [80, 81, 82]. These efforts are needed to determine the practical performance limits of spectral‐router‐based snapshot imaging systems.

## 5 Conclusion

We investigated how routing efficiency scales with channel count in inverse‐designed spectral routers for compact snapshot spectral imaging. Using adjoint‐based topology optimization and three‐dimensional FDTD simulations, we designed TiO_2_/SiO_2_ routers with 9, 16, 25, and 36 channels across the visible spectrum. The average routing efficiency increased with sub‐pixel size and approached a plateau, whereas the maximum near‐plateau efficiency decreased monotonically from 97.0% for 9 channels to 82.3% for 36 channels. Compressing the wavelength spacing to Δλ=8nm or rearranging the wavelength‐to‐sub‐pixel assignment changed the efficiency by less than 1 percentage point. Within the investigated design regime, the observed degradation is therefore associated primarily with the number of simultaneously imposed wavelength‐routing constraints rather than with wavelength spacing or local wavelength arrangement. Together with the delay‐bandwidth analysis, these results show that increasing spectral sampling in a compact finite‐thickness router results in a measurable loss of optical throughput. Therefore, practical designs must balance channel count, sub‐pixel size, device thickness, and operating bandwidth according to the spectral resolution and photon efficiency required by the imaging system.

## Author Contributions

**Junseo Han:** conceptualization, methodology, software, investigation, validation, formal analysis, visualization, writing – original draft, writing – review and editing. **Seunghyun Lee:** validation, formal analysis, writing – review and editing, writing – original draft, methodology, conceptualization, visualization. **Donghyun Kim:** software, data curation, formal analysis. **Haejun Chung:** conceptualization, funding acquisition, project administration, resources, writing – review and editing.

## Conflicts of Interest

The authors declare no conflicts of interest.

## Supporting information


Supporting Information S1

## Data Availability

The data that support the findings of this study are openly available in GitHub repository at https://github.com/nanophotonics‐lab/Spectral_Router.
