# Supplementary material for: Scaling Limits of Multichannel Spectral Routers for Snapshot Imaging
Source: Nanophotonics. 2026 Nov 13;15(22):e70360. doi: 10.1002/nap2.70360 (PMC13764598; doi:10.1002/nap2.70360)
Supplement: Supplementary file 1 [file NAP2-15-e70360-s001.pdf]

Supporting Information: Scaling Limits of Multichannel Spectral Routers for Snapshot Imaging

Junseo Han† *Seunghyun Lee*† *Donghyun Kim* *Haejun Chung**

J. Han
Department of Artificial Intelligence Semiconductor Engineering,
Hanyang University, Seoul 04763, Republic of Korea
S. Lee, D. Kim
Department of Electronic Engineering,
Hanyang University, Seoul 04763, Republic of Korea
H. Chung
Department of Artificial Intelligence Semiconductor Engineering,
Hanyang University, Seoul 04763, Republic of Korea
Department of Electronic Engineering,
Hanyang University, Seoul 04763, Republic of Korea
Email Address: haejun@hanyang.ac.kr
†These authors contributed equally to this work.

S1 Optimization Flow

The spectral routers were optimized using three-dimensional finite-difference time-domain (FDTD) simulations implemented in Meep together with the `meep.adjoint` framework. The structures were illuminated by normally incident plane waves. Periodic boundary conditions were applied in the lateral directions, and perfectly matched layers (PMLs) were used along the propagation direction.
The design region was parameterized as a TiO_2/SiO_2 material grid. Wavelength-independent refractive indices were used over the visible band:

$$n_{\mathrm{TiO_2}} = 2.65, \qquad n_{\mathrm{SiO_2}} = 1.45, \qquad n_{\mathrm{air}} = 1.0.$$

The corresponding design-material index contrast is $\Delta n = n_{\mathrm{TiO_2}} - n_{\mathrm{SiO_2}} = 1.20$. Unless otherwise stated, the device thickness was fixed at $h = 2\,\mu\mathrm{m}$. For an $N = k \times k$-channel router, the super-pixel side length was kP, where P is the sub-pixel size. The sub-pixel size was swept over eight values from $0.12\,\mu\mathrm{m}$ to $0.60\,\mu\mathrm{m}$.
For each wavelength channel, the per-channel routing efficiency $T_{ii}(\lambda_i)$ was calculated from the z-directed Poynting flux integrated over the corresponding target sub-pixel region and normalized by the incident power. During optimization, the individual channel efficiencies were combined using a negative-p-norm objective,

$$\mathrm{FoM} = \left[\frac{1}{N} \sum_{i=1}^{N} \big(T_{ii}(\lambda_i) + \epsilon \big)^p \right]^{1/p}. \tag{S1}$$

This objective serves as a differentiable soft-minimum that emphasizes the lower-efficiency channels during optimization. The average routing efficiency was then used for the final performance comparison in the main text.
The main optimization settings are summarized in Table S1. After optimization, the final binary TiO_2/SiO_2 structures were evaluated using FDTD simulations, from which the average routing efficiency and the achieved worst-channel value $\rho_{0,\min}$ were extracted.

Table S1: Optimization settings used for the spectral-router designs.

Parameter	Value
FDTD resolution	50 pixels/µm
Boundary condition	Periodic in x, y, with PML in z
Device thickness	2 µm
Sub-pixel size sweep	0.12 µm–0.60 µm
Design materials	TiO_2 / SiO_2
Refractive indices	$n_{TiO_2} = 2.65,\ n_{SiO_2} = 1.45$
FoM exponent p	-4
FoM offset ϵ	10^{-6}
Optimizer	Adam (custom)
Learning rate	0.1
Adam $\beta_1, \beta_2, \epsilon_{Adam}$	$0.9,\ 0.999,\ 10^{-8}$
Warm-up iterations	10
Total iterations	200
Initialization	Uniform random on $[0.45, 0.55]$
Minimum feature size	20 nm
Projection sharpness β	$1 \to 32$ (exponential)

Multiple random initializations were used selectively. For some configurations, additional runs with different random seeds were performed to seek better balance among channel efficiencies. Each additional run used the same optimization settings and iteration count as the corresponding initial run. The number of runs therefore varied among configurations. These additional runs do not guarantee that the reported designs are globally optimal.

S2 Target Wavelength Sets and Channel Layouts

The target wavelength sets used for the channel-count scaling study are summarized in Table S2. For each $N = k \times k$ router, the wavelengths were assigned to a $k \times k$ sub-pixel array in row-major order from shorter to longer wavelengths. Each entry in the table denotes the target wavelength assigned to the corresponding sub-pixel position.

Table S2: Target-wavelength layouts used for the channel-count scaling study. Entries are arranged according to the $k \times k$ sub-pixel array for each $N = k \times k$ router. Each value denotes the target wavelength in nm.

N	Row	1	2	3	4	5	6
9	1	430	460	490			
	2	520	550	580			
	3	610	640	670			
16	1	410	430	450	470		
	2	490	510	525	540		
	3	560	575	590	610		
	4	630	650	670	690		
25	1	406	418	430	442	454	
	2	466	478	490	502	514	
	3	526	538	550	562	574	
	4	586	598	610	622	634	
	5	646	658	670	682	694	
36	1	404	413	421	429	438	446
	2	454	463	471	479	488	496
	3	504	513	521	529	538	546
	4	554	563	571	579	588	596
	5	604	613	621	629	638	646
	6	654	663	671	679	688	696

The compressed-spacing control configurations used in Fig. 4 of the main text reassign the same spatial layouts to a nominal $\Delta\lambda = 8\,\text{nm}$ sampling, evaluated at $P = 0.34\,\mu\text{m}$. The corresponding target-wavelength layouts are listed in Table S3. Because the target wavelengths are rounded to integer nanometer values, the realized spacings mix 8 nm and 9 nm steps.

Table S3: Compressed-spacing target-wavelength layouts used for the wavelength-spacing control (nominal $\Delta\lambda = 8\,\text{nm}$). Entries follow the same row-major convention as Table S2, each value denotes the target wavelength in nm.

N	Row	1	2	3	4	5
9	1	513	521	529		
	2	538	546	554		
	3	563	571	579		
16	1	480	488	497	505	
	2	513	522	530	538	
	3	547	555	563	572	
	4	580	588	597	605	
25	1	454	463	471	479	488
	2	496	504	513	521	529
	3	538	546	554	563	571
	4	579	588	596	604	613
	5	621	629	638	646	654

S3 Construction of the Wavelength Arrangement

This section documents the construction of the $\overline{\Delta\lambda}_{\text{adj}}$-maximized arrangement compared in Fig. 5 of the main text. The 36 channels are indexed $k = 0, \ldots, 35$ in spectral order, following the row-major layout of Table S2, and the sub-pixel positions $p = 0, \ldots, 35$ are indexed in the same row-major order. The re-arranged layout assigns channel k to position

$$p(k) = (s\,k) \bmod 36, \qquad s = 19. \tag{S2}$$

Because $\gcd(s, 36) = 1$, this map is a valid permutation of the 36 sub-pixels. Since $19^2 \equiv 1 \pmod{36}$, the permutation is an involution. Equivalently, channels with even spectral indices satisfy $p(k) = k$, whereas channels with odd spectral indices are shifted by 18 row-major positions modulo 36.
The step s was selected by evaluating, for every s coprime to 36, the average absolute wavelength difference $\overline{\Delta\lambda}_{\text{adj}}$ between each sub-pixel and its four nearest neighbors, with periodic boundary conditions consistent with the laterally periodic simulation domain (Table S4). The sequential arrangement ($s = 1$) yields $\overline{\Delta\lambda}_{\text{adj}} = 48.67\,\text{nm}$, and $s = 19$ maximizes the separation with $\overline{\Delta\lambda}_{\text{adj}} = 116.67\,\text{nm}$, corresponding to the two values reported in Fig. 5 of the main text. The resulting target-wavelength layout is listed in Table S5.

Table S4: Average adjacent-sub-pixel wavelength difference $\overline{\Delta\lambda}_{\text{adj}}$ for all modular steps s coprime to 36.

s	$\overline{\Delta\lambda}_{\text{adj}}$ (nm)
1 (sequential)	48.67
5	81.97
7	83.33
11	106.97
13	108.33
17	115.31
19 (selected)	116.67
23	106.97
25	108.33
29	81.97
31	83.33
35	55.47

Table S5: Target-wavelength layout of the $\overline{\Delta\lambda}_{\text{adj}}$-maximized ($s = 19$) arrangement. Entries follow the same row-major convention as Table S2, each value denotes the target wavelength in nm.

Row	1	2	3	4	5	6
1	404	563	421	579	438	596
2	454	613	471	629	488	646
3	504	663	521	679	538	696
4	554	413	571	429	588	446
5	604	463	621	479	638	496
6	654	513	671	529	688	546

S4 Sub-Pixel-Size Sweep: 9-Channel Router

Supplementary S4–S7 present the complete sub-pixel-size sweep data underlying Fig. 3(d) of the main text, with one section per channel count. For each sub-pixel size P, the three-dimensional layout of the optimized design region, the focal-plane S_z montage, and the corresponding routing-efficiency spectra are shown. Each montage tile shows the focal-plane S_z map over the full super-pixel at the corresponding target wavelength, labeled following the channel layouts of Table S2. The white box in each map indicates the target sub-pixel region, and each map is normalized independently for visualization. The routing efficiency of each channel is evaluated at its target wavelength.
Figure S1 shows the three-dimensional design layouts of the 9-channel router for all eight sub-pixel sizes, Fig. S2 shows the focal-plane S_z montages, and Fig. S3 shows the corresponding routing-efficiency spectra.

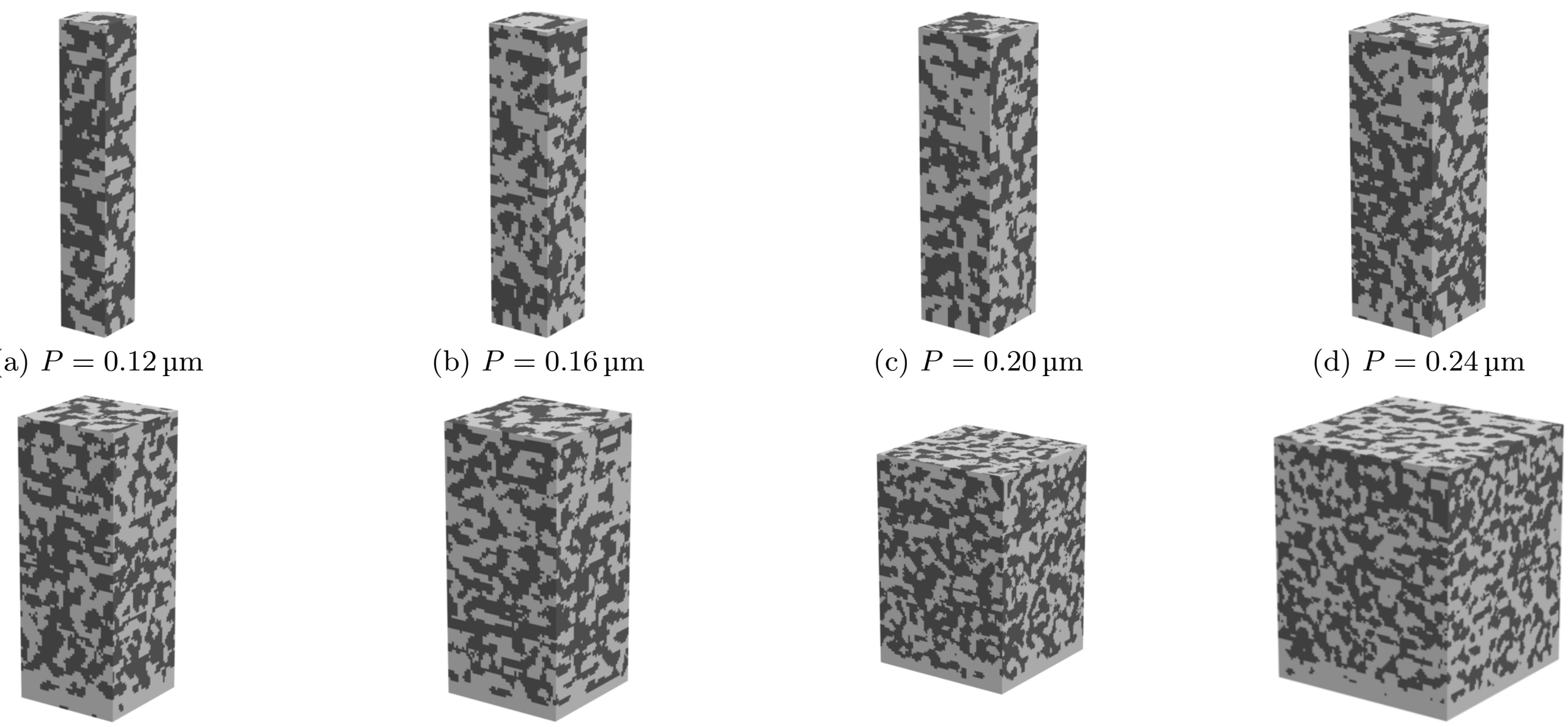

(a) $P = 0.12\,\mu m$ (b) $P = 0.16\,\mu m$ (c) $P = 0.20\,\mu m$ (d) $P = 0.24\,\mu m$

(e) $P = 0.28\,\mu m$ (f) $P = 0.34\,\mu m$ (g) $P = 0.50\,\mu m$ (h) $P = 0.60\,\mu m$

Figure S1: Three-dimensional layouts of the optimized 9-channel design regions across the sub-pixel-size sweep, $P = 0.12\,\mu m$ to $0.60\,\mu m$, at $h = 2\,\mu m$. The light gray corresponds to SiO_2 and the dark gray to TiO_2.

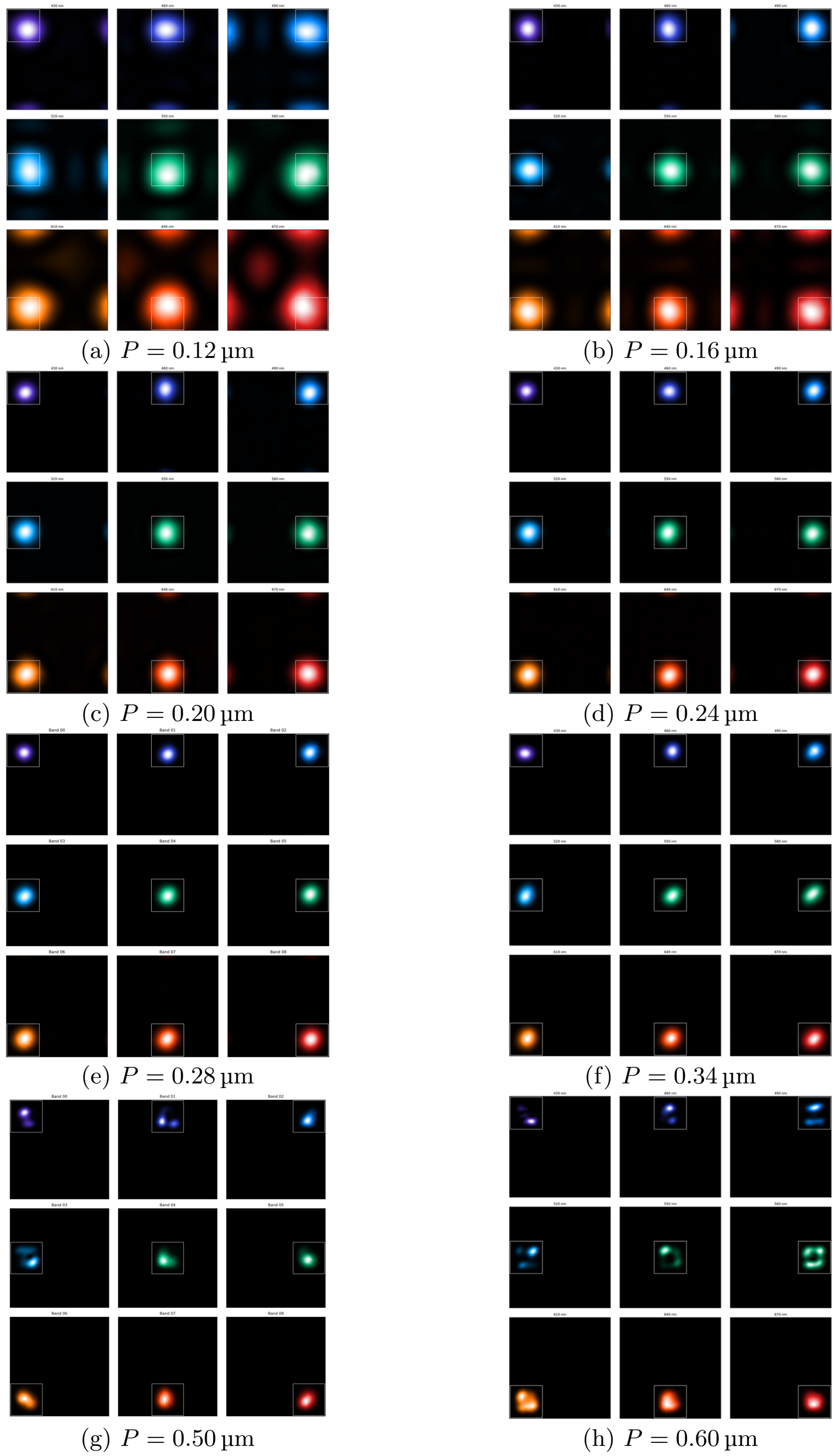

(a) $P = 0.12\,\mu\text{m}$ (b) $P = 0.16\,\mu\text{m}$ (c) $P = 0.20\,\mu\text{m}$ (d) $P = 0.24\,\mu\text{m}$ (e) $P = 0.28\,\mu\text{m}$ (f) $P = 0.34\,\mu\text{m}$ (g) $P = 0.50\,\mu\text{m}$ (h) $P = 0.60\,\mu\text{m}$

Figure S2: Focal-plane S_z montages of the 9-channel router across the sub-pixel-size sweep, $P = 0.12\,\mu\text{m}$ to $0.60\,\mu\text{m}$. The white box in each channel map indicates the target sub-pixel region, and each map is normalized independently for visualization.

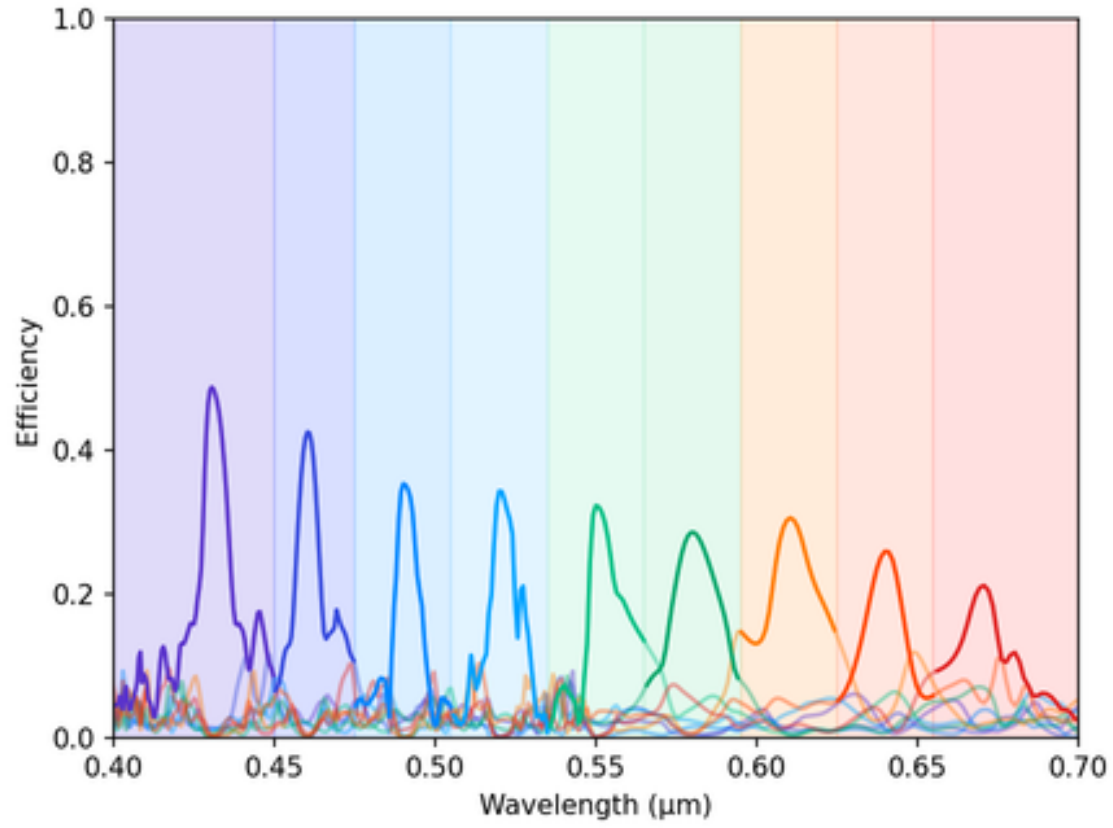

(a) $P = 0.12\,\mu m$

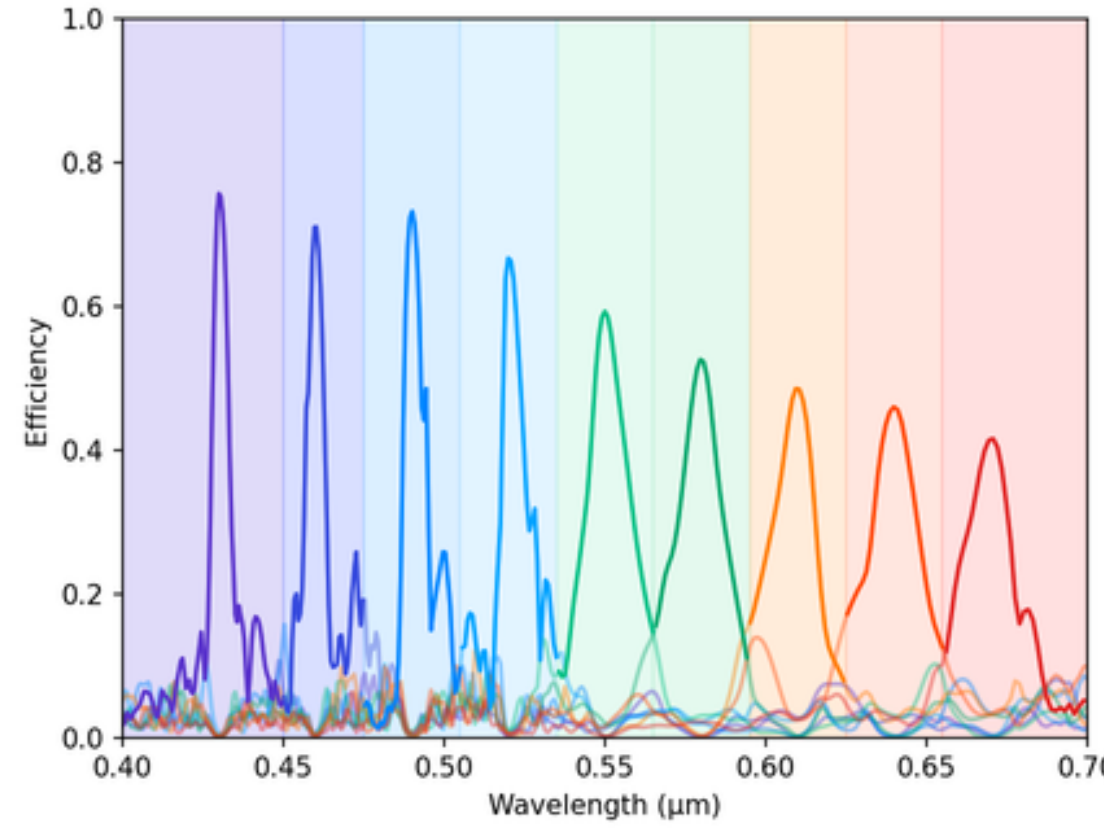

(b) $P = 0.16\,\mu m$

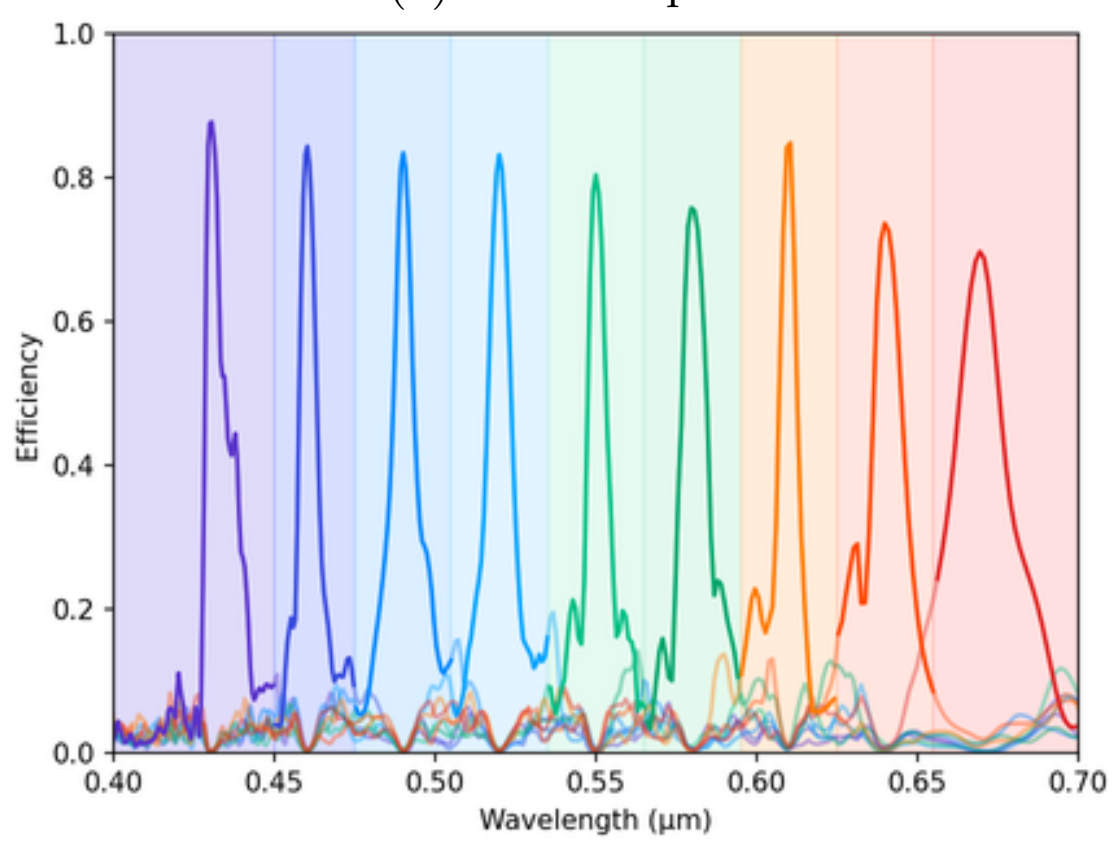

(c) $P = 0.20\,\mu m$

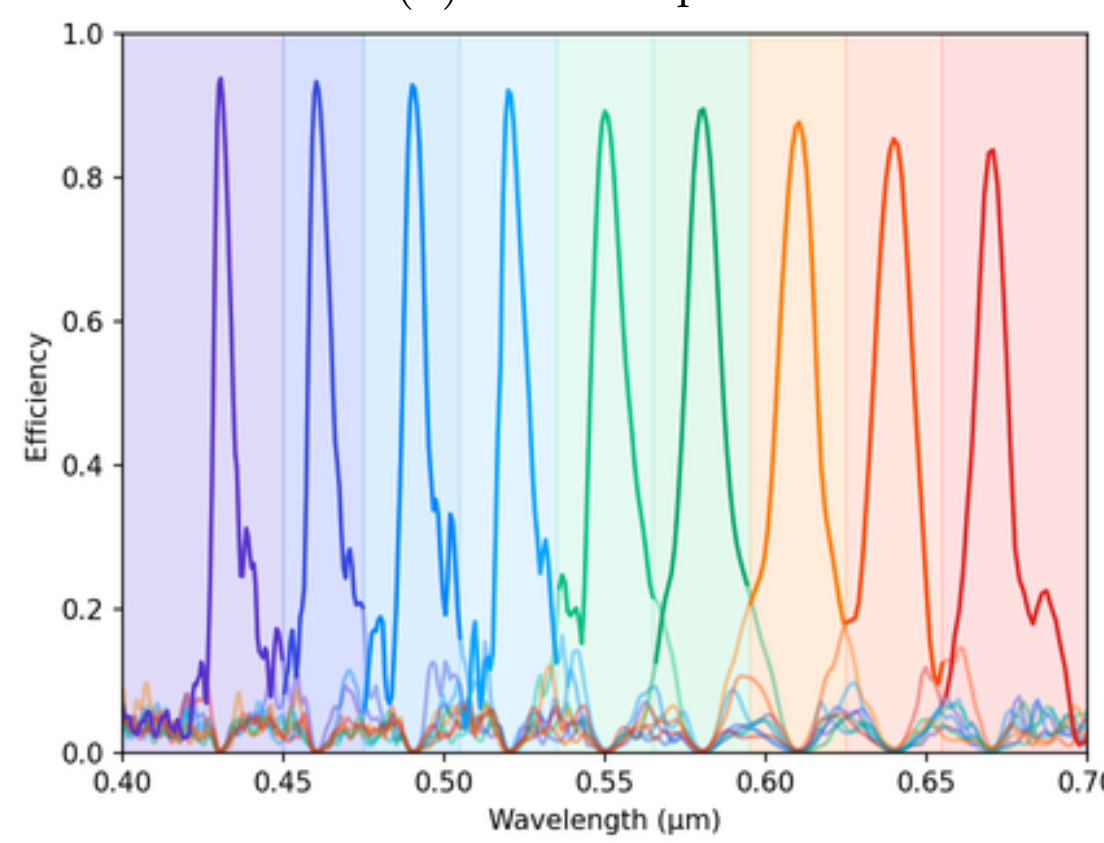

(d) $P = 0.24\,\mu m$

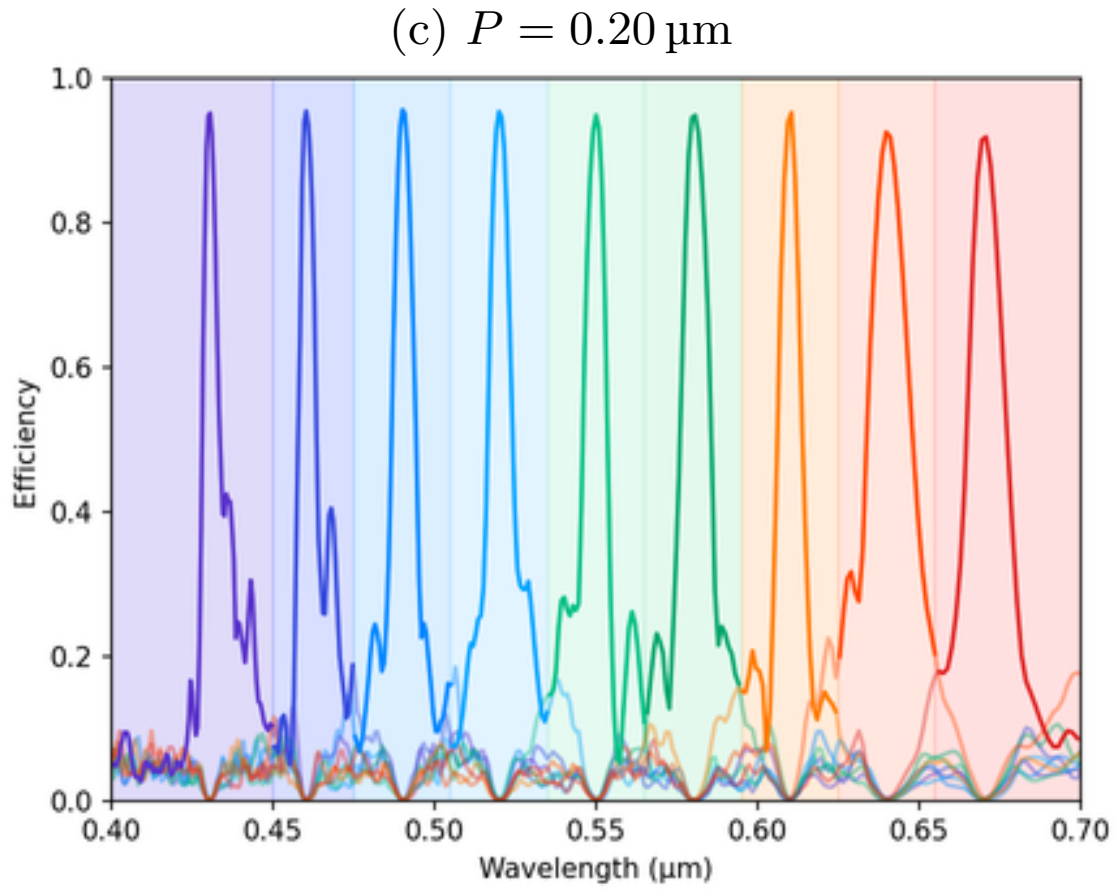

(e) $P = 0.28\,\mu m$

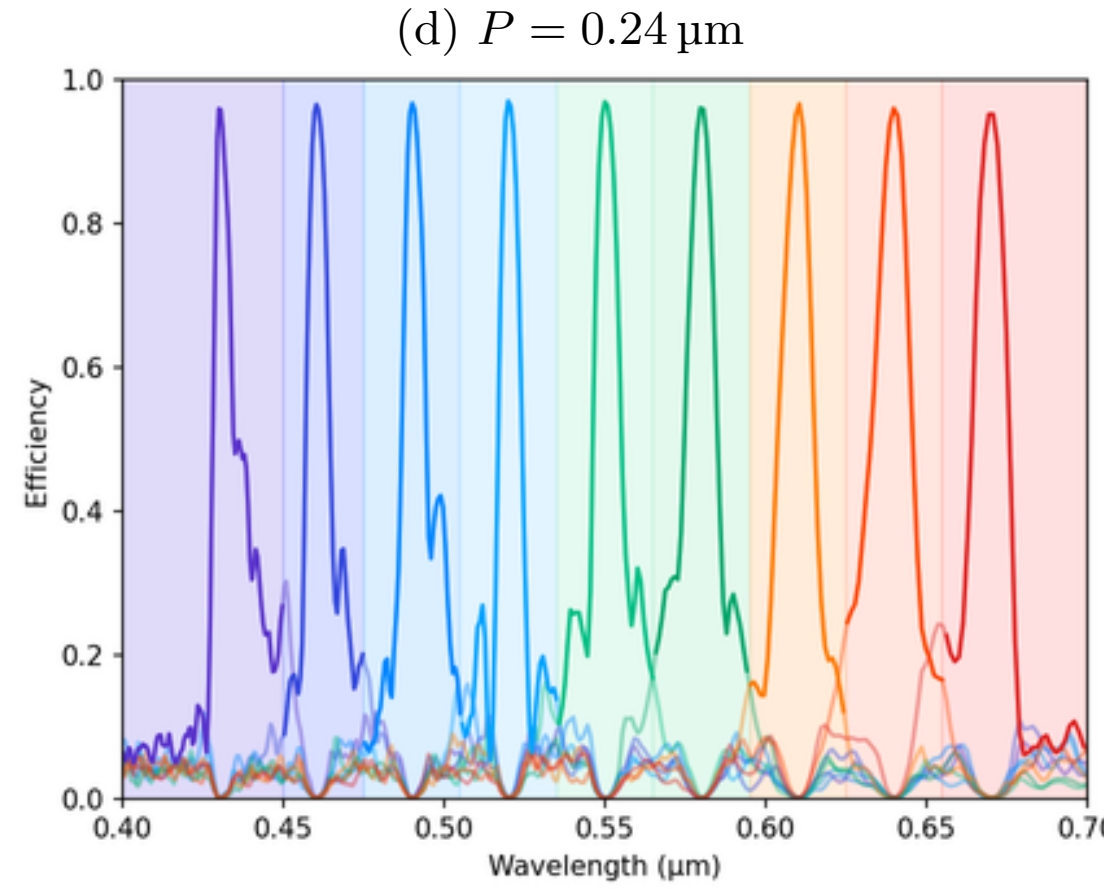

(f) $P = 0.34\,\mu m$

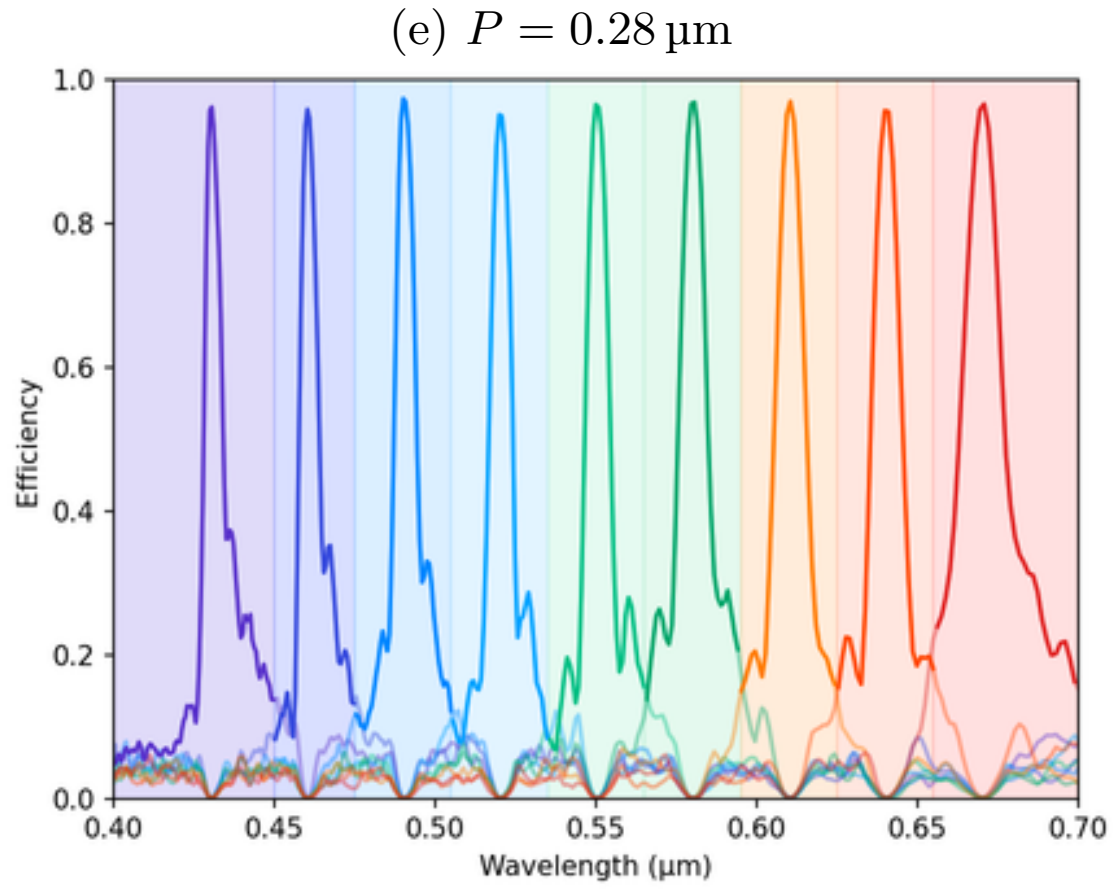

(g) $P = 0.50\,\mu m$

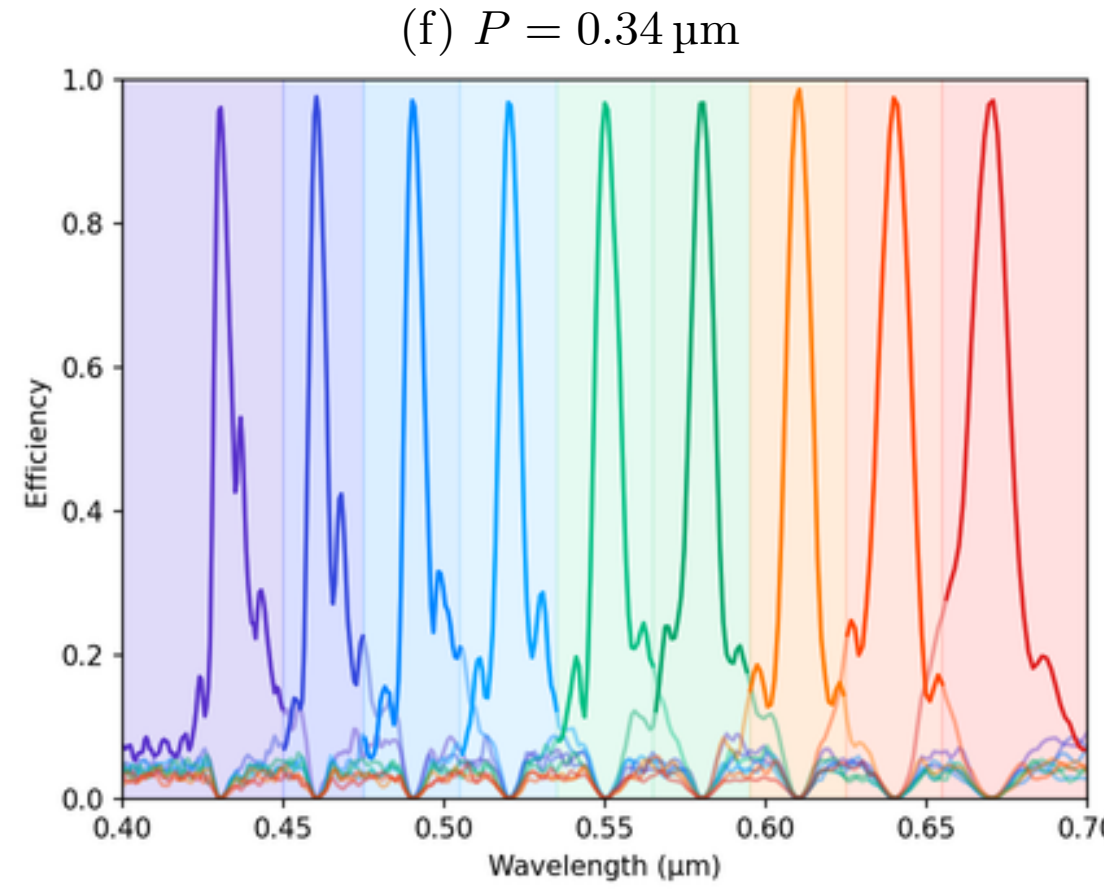

(h) $P = 0.60\,\mu m$

Figure S3: Routing-efficiency spectra of the 9-channel router across the sub-pixel-size sweep. The routing efficiency of each channel is evaluated at its target wavelength.

S5 Sub-Pixel-Size Sweep: 16-Channel Router

Figures S4–S6 present the corresponding sweep data for the 16-channel router. Panel conventions follow Supplementary S4.

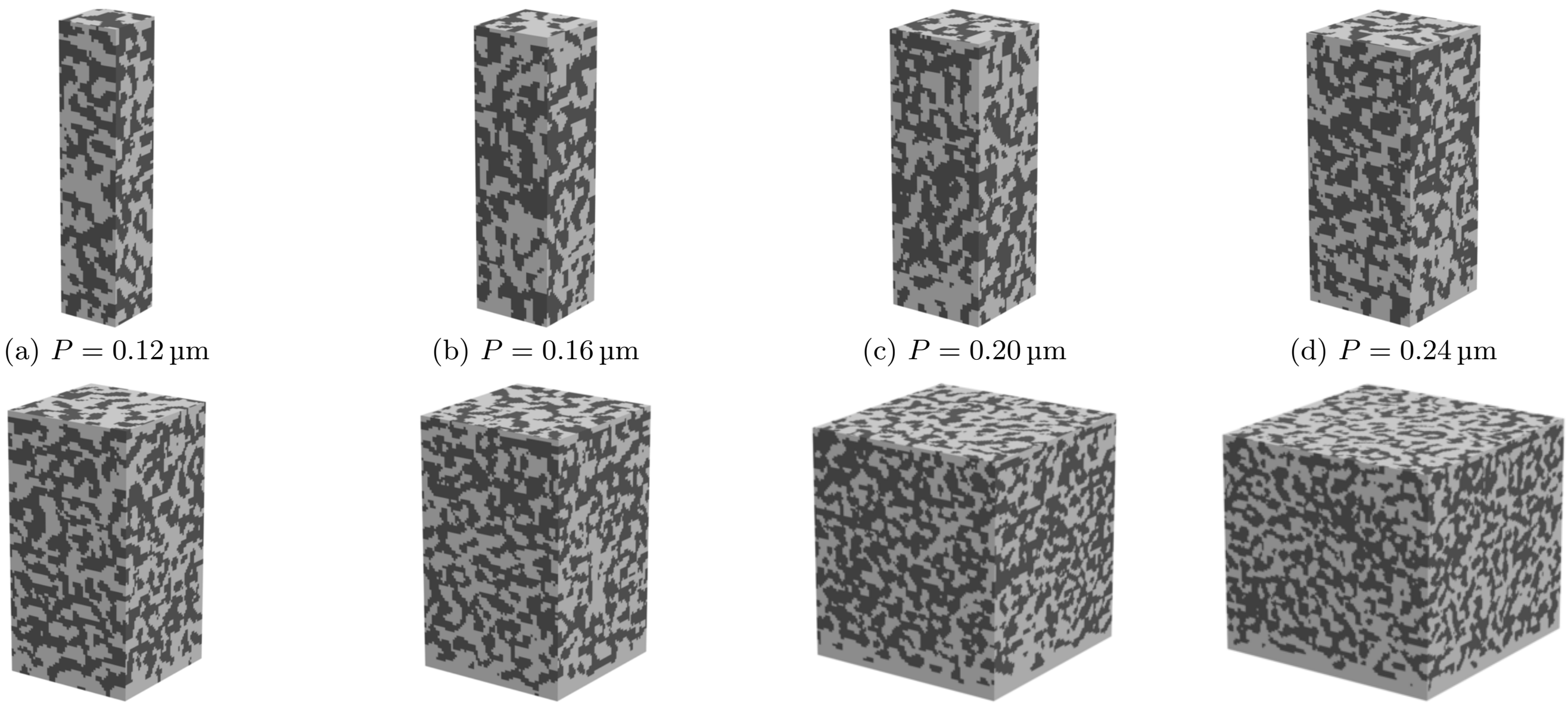

(a) $P = 0.12\,\mu m$ (b) $P = 0.16\,\mu m$ (c) $P = 0.20\,\mu m$ (d) $P = 0.24\,\mu m$

(e) $P = 0.28\,\mu m$ (f) $P = 0.34\,\mu m$ (g) $P = 0.50\,\mu m$ (h) $P = 0.60\,\mu m$

Figure S4: Three-dimensional layouts of the optimized 16-channel design regions across the sub-pixel-size sweep, $P = 0.12\,\mu m$ to $0.60\,\mu m$, at $h = 2\,\mu m$. The light gray corresponds to SiO_2 and the dark gray to TiO_2.

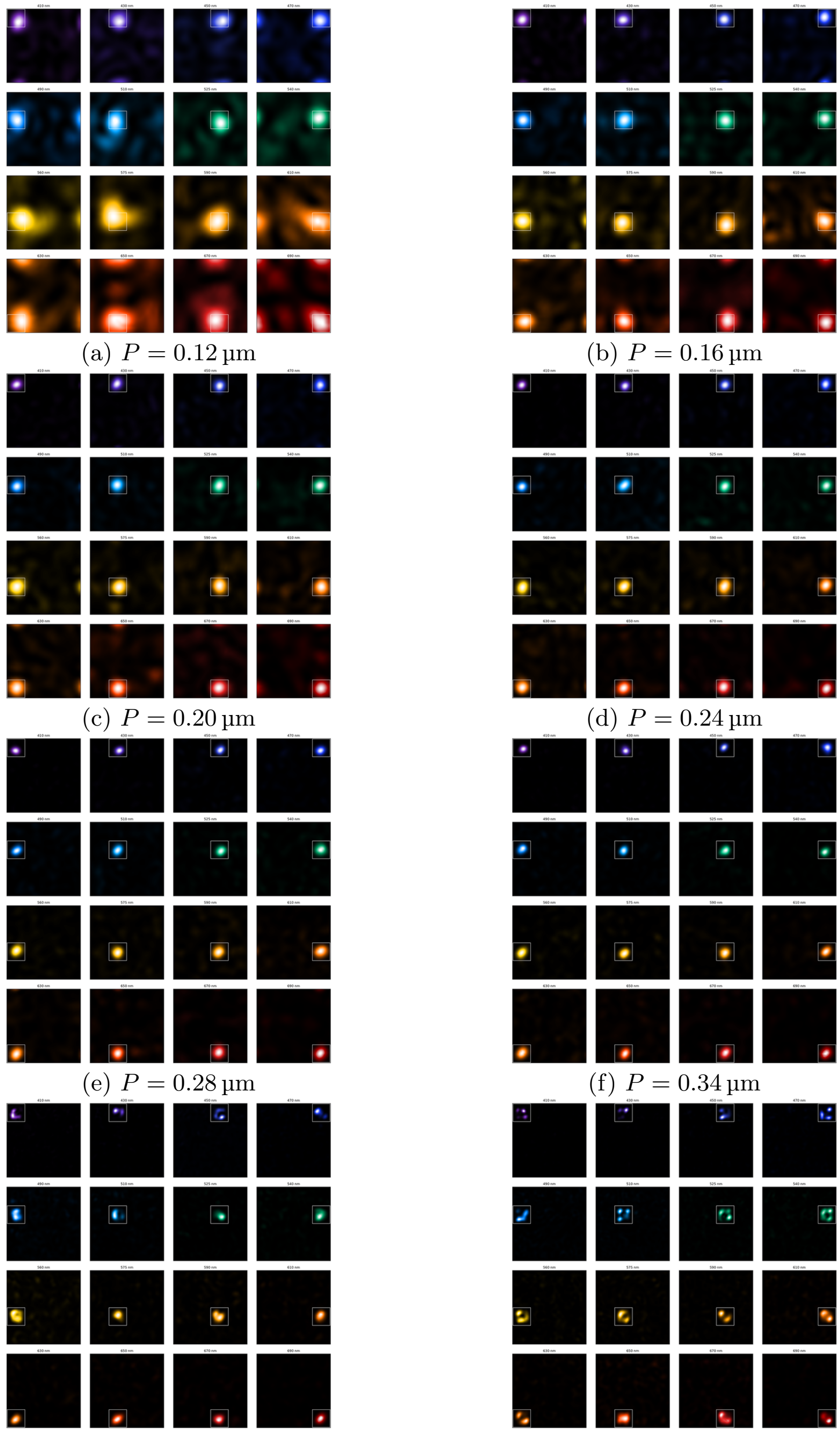

Figure S5: Focal-plane S_z montages of the 16-channel router across the sub-pixel-size sweep, $P = 0.12\,\mu m$ to $0.60\,\mu m$. The white box in each channel map indicates the target sub-pixel region, and each map is normalized independently for visualization.

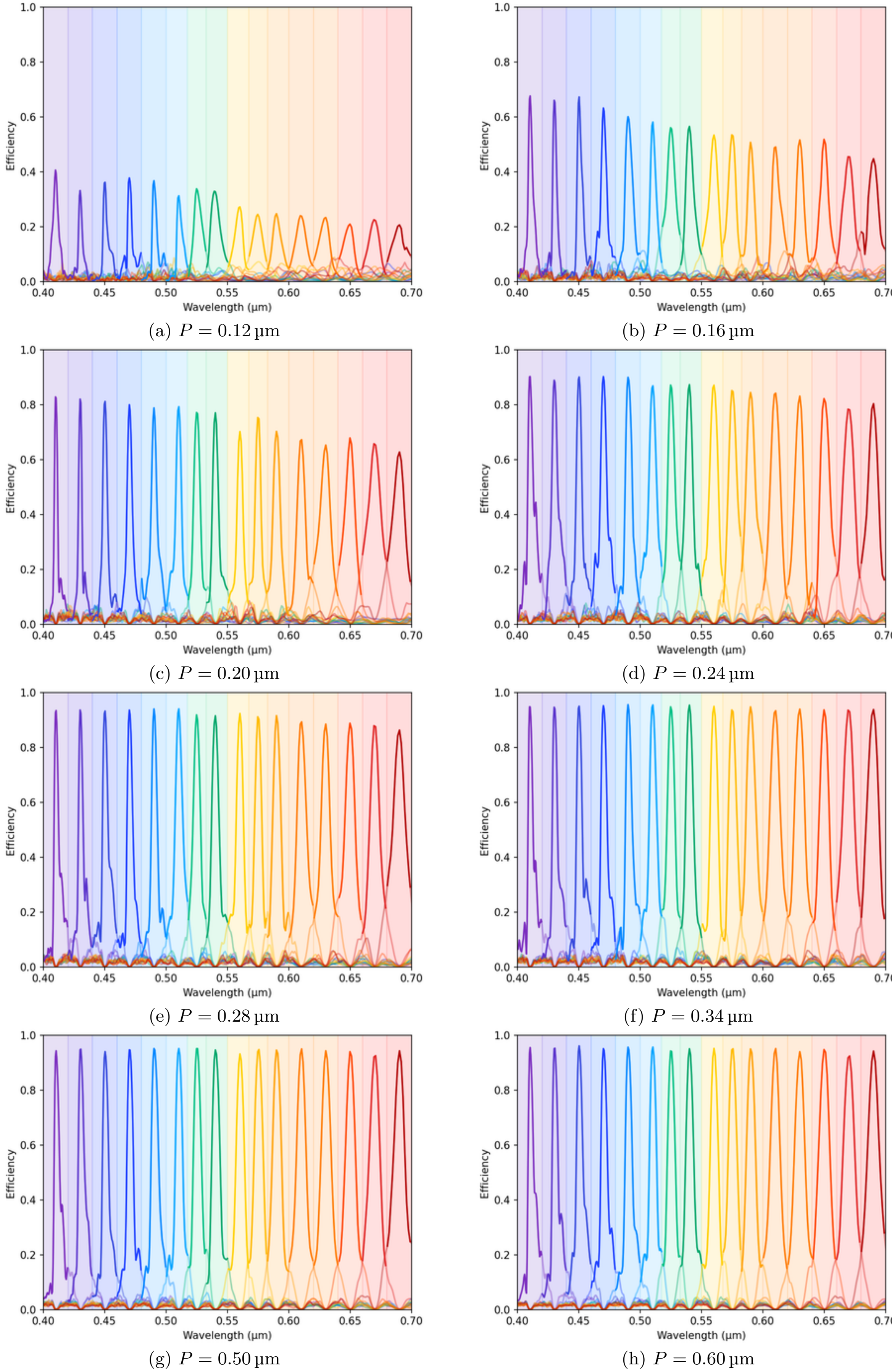

(a) $P = 0.12\,\mu\mathrm{m}$ (b) $P = 0.16\,\mu\mathrm{m}$

(c) $P = 0.20\,\mu\mathrm{m}$ (d) $P = 0.24\,\mu\mathrm{m}$

(e) $P = 0.28\,\mu\mathrm{m}$ (f) $P = 0.34\,\mu\mathrm{m}$

(g) $P = 0.50\,\mu\mathrm{m}$ (h) $P = 0.60\,\mu\mathrm{m}$

Figure S6: Routing-efficiency spectra of the 16-channel router across the sub-pixel-size sweep. The routing efficiency of each channel is evaluated at its target wavelength.

S6 Sub-Pixel-Size Sweep: 25-Channel Router

Figures S7–S9 present the corresponding sweep data for the 25-channel router. Panel conventions follow Supplementary S4.

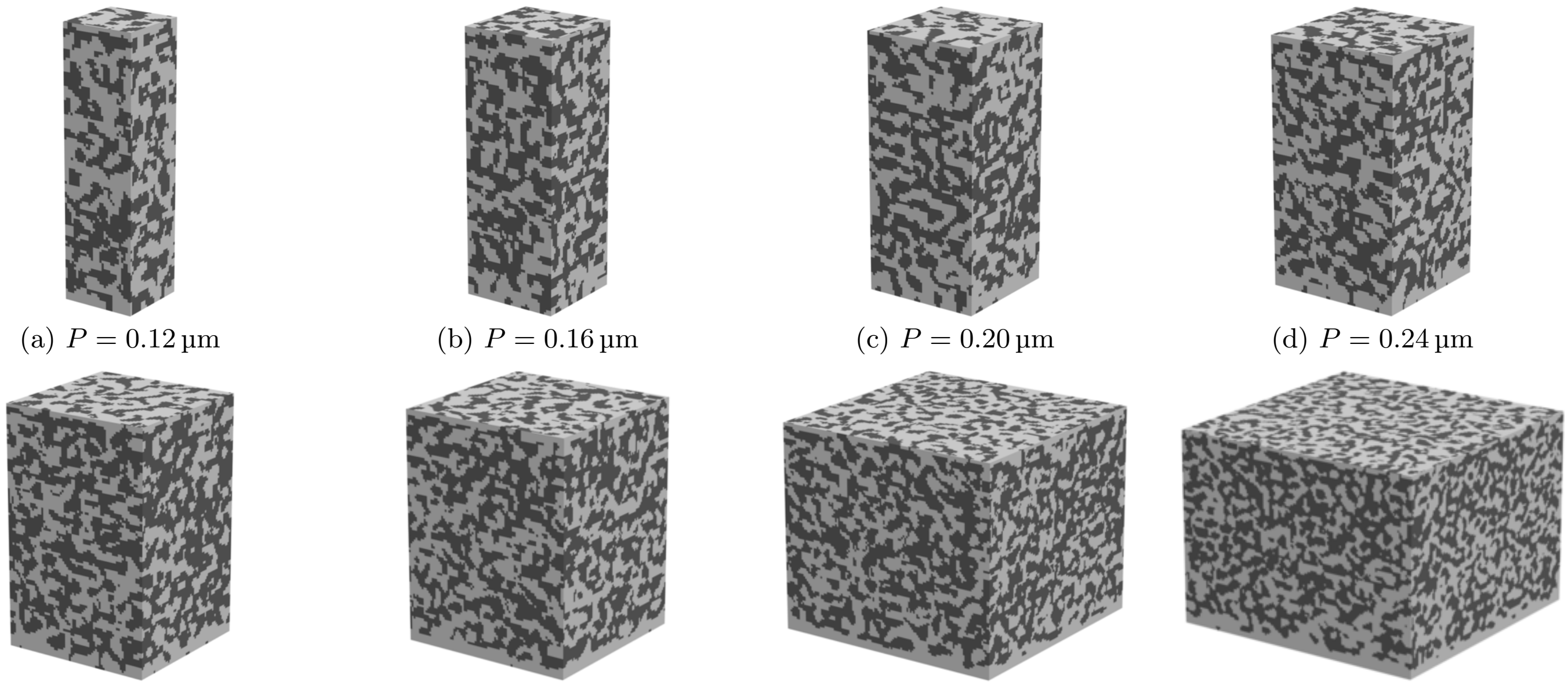

(a) $P = 0.12\,\mu\text{m}$ (b) $P = 0.16\,\mu\text{m}$ (c) $P = 0.20\,\mu\text{m}$ (d) $P = 0.24\,\mu\text{m}$

(e) $P = 0.28\,\mu\text{m}$ (f) $P = 0.34\,\mu\text{m}$ (g) $P = 0.50\,\mu\text{m}$ (h) $P = 0.60\,\mu\text{m}$

Figure S7: Three-dimensional layouts of the optimized 25-channel design regions across the sub-pixel-size sweep, $P = 0.12\,\mu\text{m}$ to $0.60\,\mu\text{m}$, at $h = 2\,\mu\text{m}$. The light gray corresponds to SiO_2 and the dark gray to TiO_2.

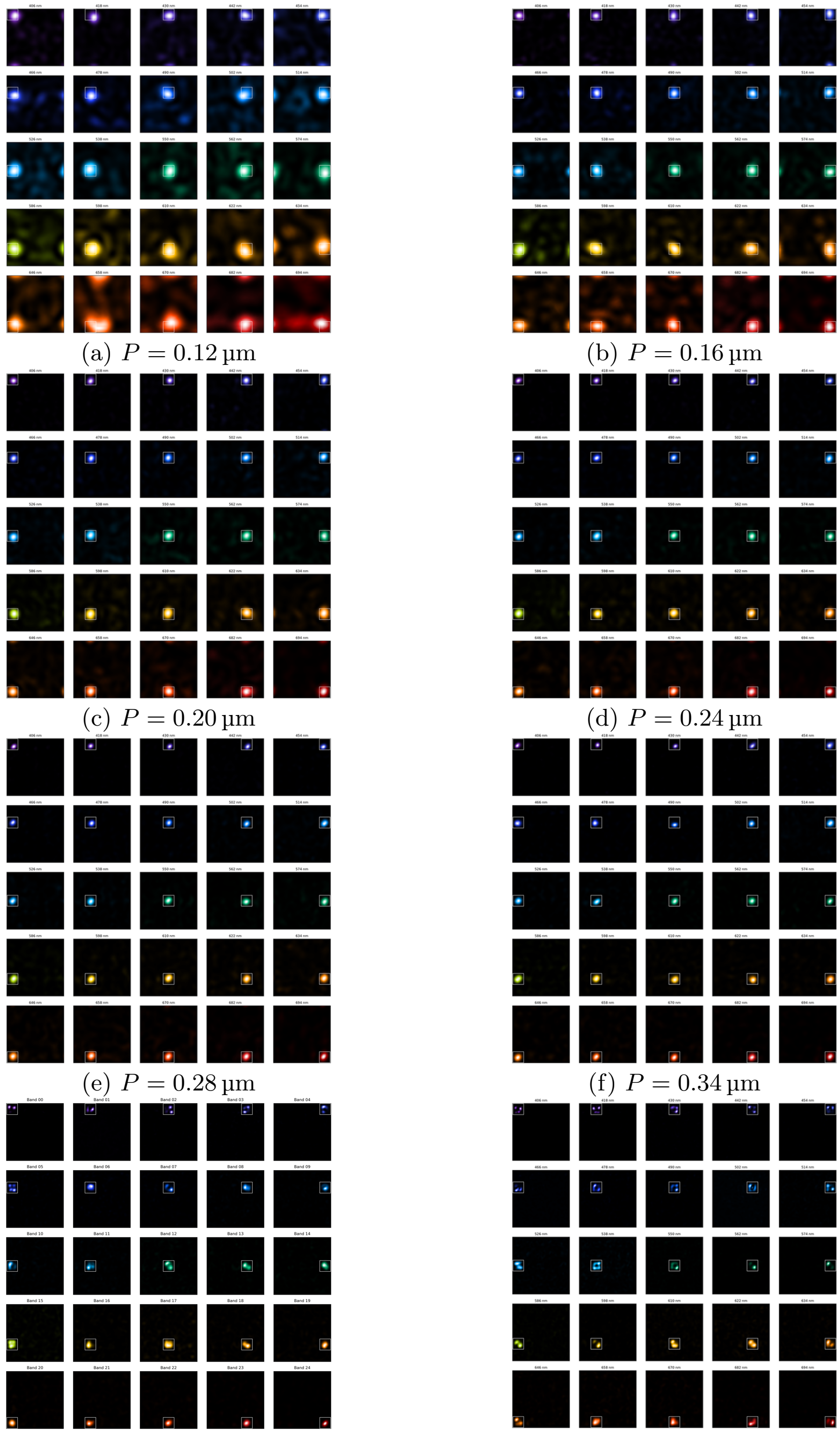

(a) $P = 0.12\,\mu m$
(b) $P = 0.16\,\mu m$
(c) $P = 0.20\,\mu m$
(d) $P = 0.24\,\mu m$
(e) $P = 0.28\,\mu m$
(f) $P = 0.34\,\mu m$
(g) $P = 0.50\,\mu m$
(h) $P = 0.60\,\mu m$

Figure S8: Focal-plane S_z montages of the 25-channel router across the sub-pixel-size sweep, $P = 0.12\,\mu m$ to $0.60\,\mu m$. The white box in each channel map indicates the target sub-pixel region, and each map is normalized independently for visualization.

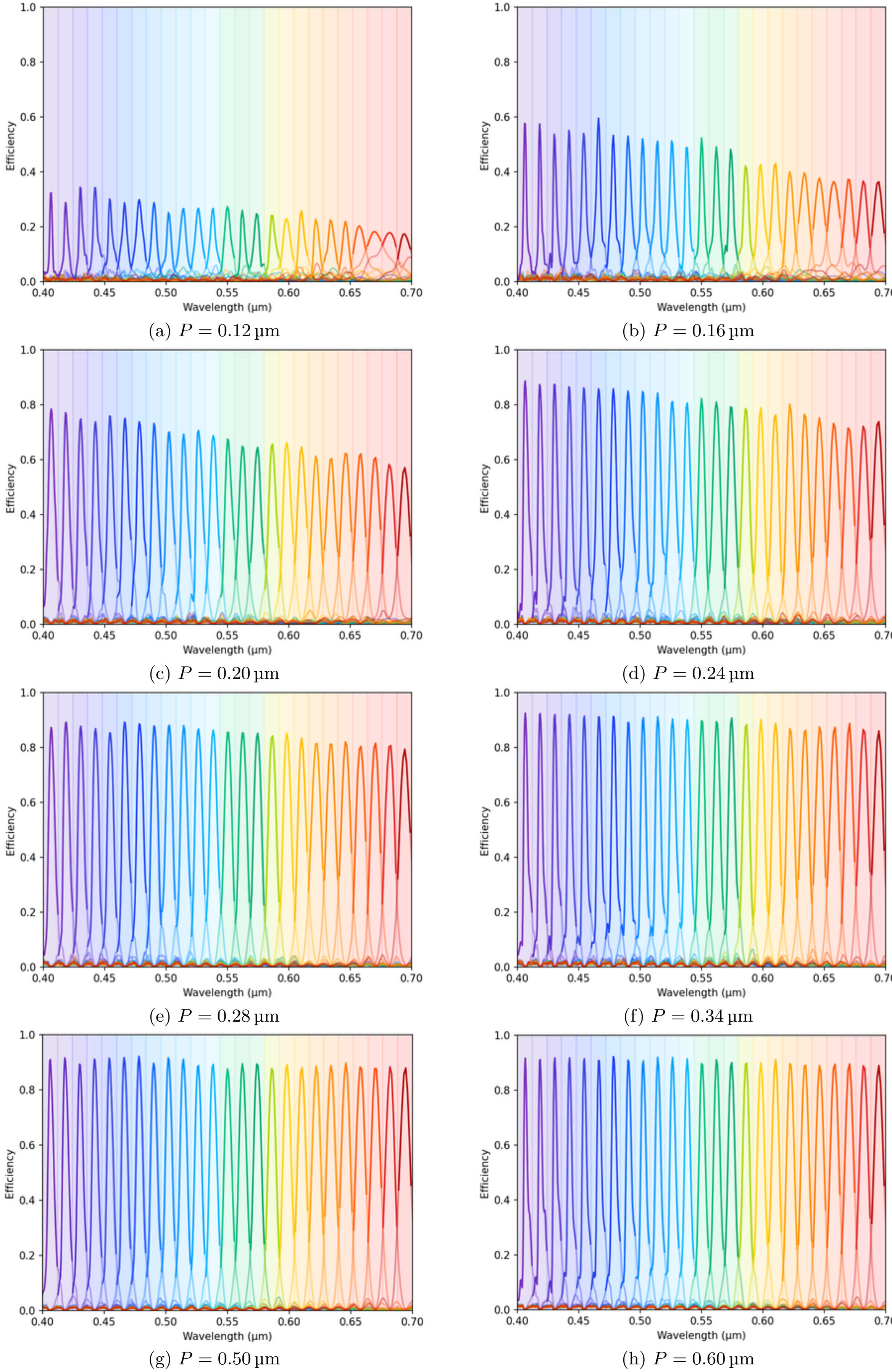

(a) $P = 0.12\,\mu m$ (b) $P = 0.16\,\mu m$ (c) $P = 0.20\,\mu m$ (d) $P = 0.24\,\mu m$ (e) $P = 0.28\,\mu m$ (f) $P = 0.34\,\mu m$ (g) $P = 0.50\,\mu m$ (h) $P = 0.60\,\mu m$

Figure S9: Routing-efficiency spectra of the 25-channel router across the sub-pixel-size sweep. The routing efficiency of each channel is evaluated at its target wavelength.

S7 Sub-Pixel-Size Sweep: 36-Channel Router

Figures S10–S12 present the corresponding sweep data for the 36-channel router. Panel conventions follow Supplementary S4.

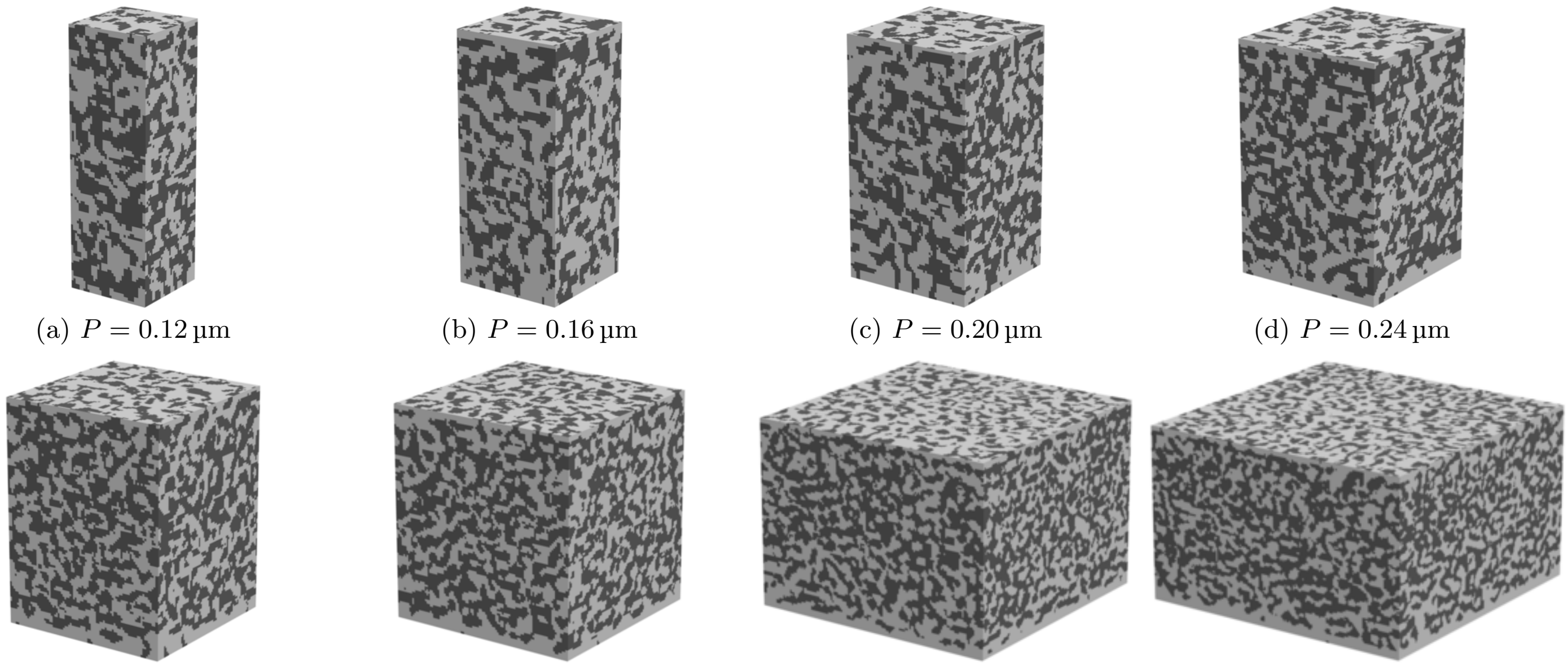

(a) $P = 0.12\,\mu m$ (b) $P = 0.16\,\mu m$ (c) $P = 0.20\,\mu m$ (d) $P = 0.24\,\mu m$

(e) $P = 0.28\,\mu m$ (f) $P = 0.34\,\mu m$ (g) $P = 0.50\,\mu m$ (h) $P = 0.60\,\mu m$

Figure S10: Three-dimensional layouts of the optimized 36-channel design regions across the sub-pixel-size sweep, $P = 0.12\,\mu m$ to $0.60\,\mu m$, at $h = 2\,\mu m$. The light gray corresponds to SiO_2 and the dark gray to TiO_2.

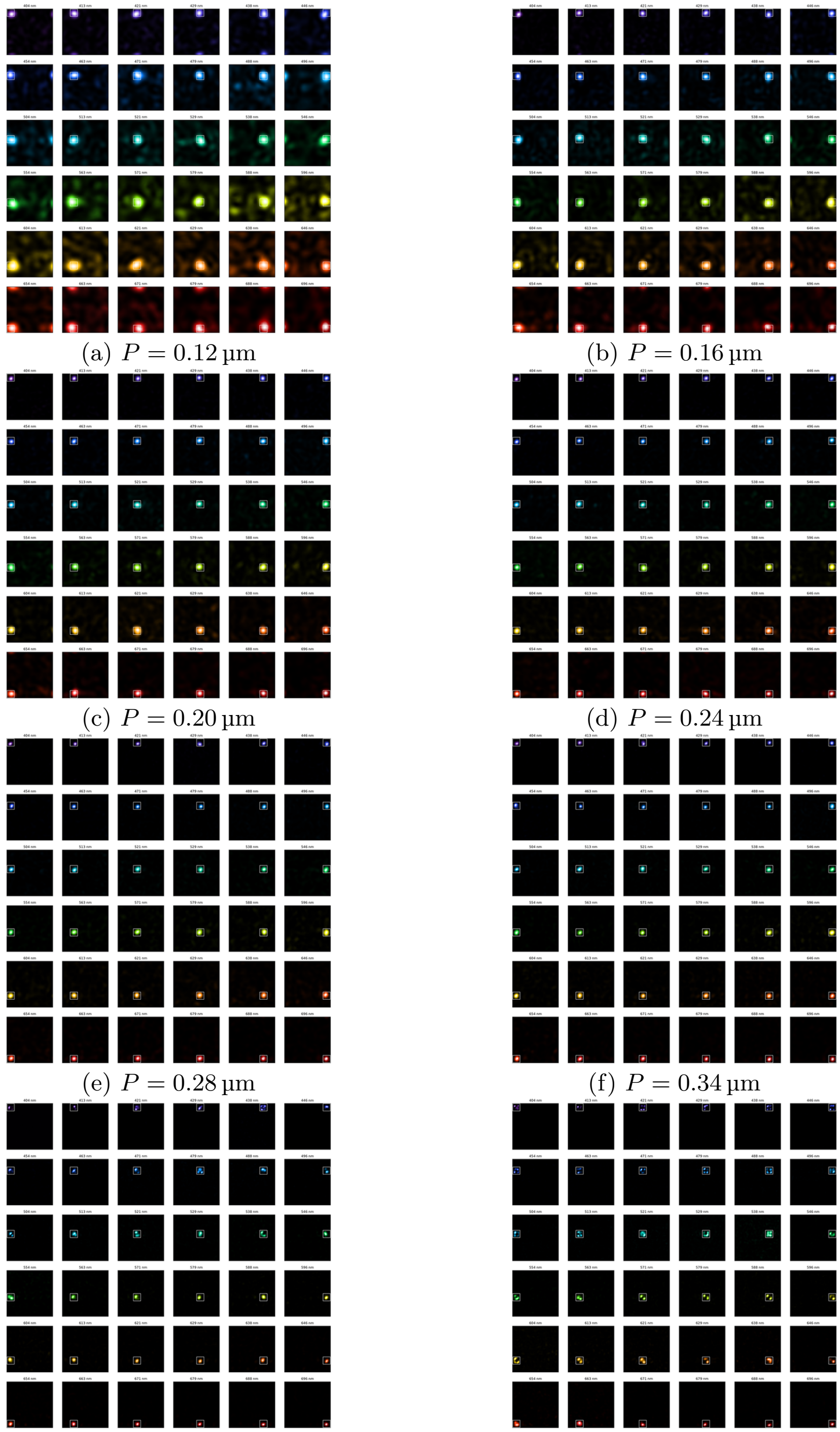

(a) $P = 0.12\,\mu\text{m}$ (b) $P = 0.16\,\mu\text{m}$ (c) $P = 0.20\,\mu\text{m}$ (d) $P = 0.24\,\mu\text{m}$ (e) $P = 0.28\,\mu\text{m}$ (f) $P = 0.34\,\mu\text{m}$ (g) $P = 0.50\,\mu\text{m}$ (h) $P = 0.60\,\mu\text{m}$

Figure S11: Focal-plane S_z montages of the 36-channel router across the sub-pixel-size sweep, $P = 0.12\,\mu\text{m}$ to $0.60\,\mu\text{m}$. The white box in each channel map indicates the target sub-pixel region, and each map is normalized independently for visualization.

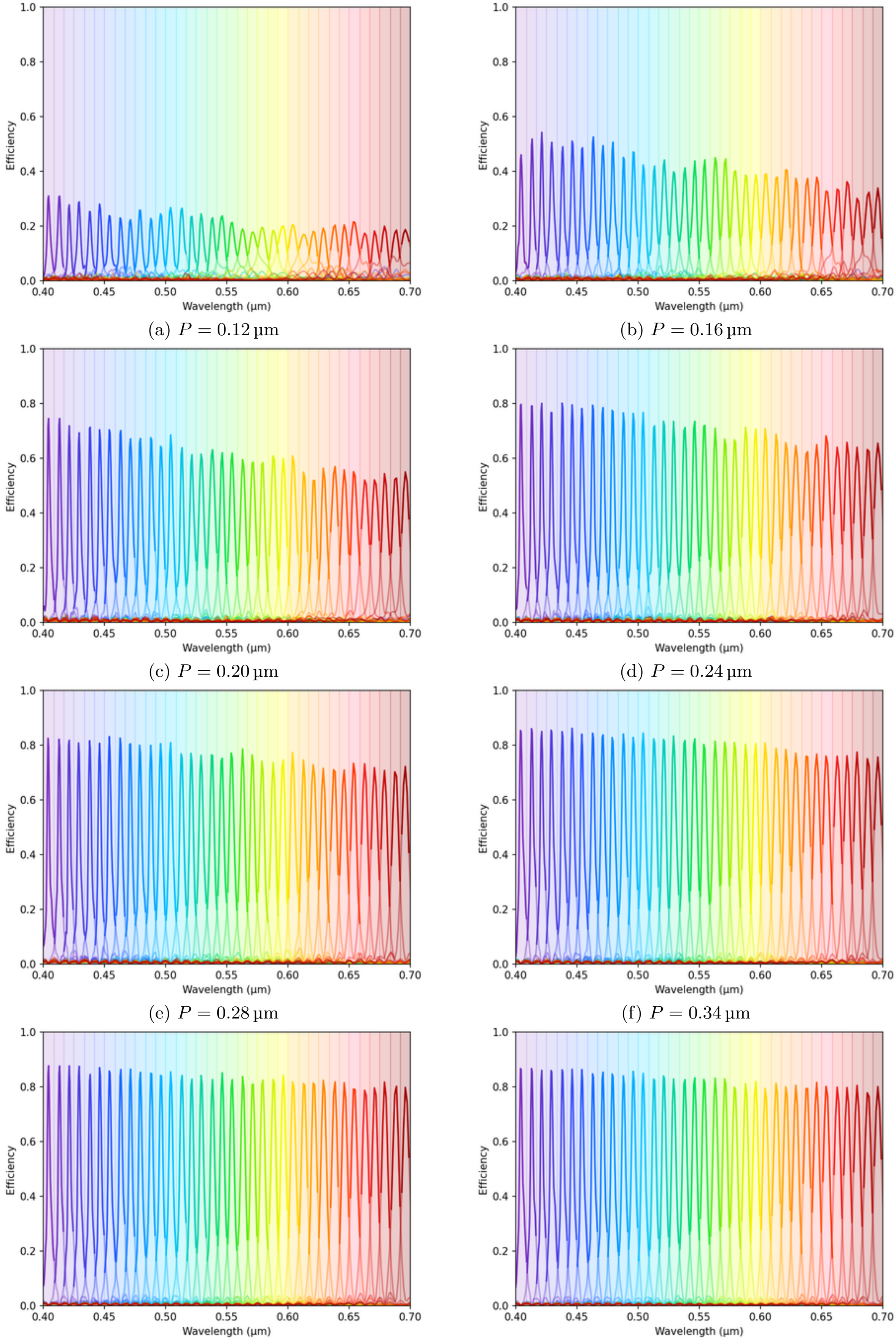

(a) $P = 0.12\,\mu\mathrm{m}$ (b) $P = 0.16\,\mu\mathrm{m}$

(c) $P = 0.20\,\mu\mathrm{m}$ (d) $P = 0.24\,\mu\mathrm{m}$

(e) $P = 0.28\,\mu\mathrm{m}$ (f) $P = 0.34\,\mu\mathrm{m}$

(g) $P = 0.50\,\mu\mathrm{m}$ (h) $P = 0.60\,\mu\mathrm{m}$

Figure S12: Routing-efficiency spectra of the 36-channel router across the sub-pixel-size sweep. The routing efficiency of each channel is evaluated at its target wavelength.

S8 Compressed-Spacing Control: Focal-Plane S_z Maps

This section presents the focal-plane S_z montages of the compressed-spacing control configurations compared in Fig. 4(d)–(f) of the main text. The corresponding target-wavelength layouts are listed in Table S3. All configurations are evaluated at $P = 0.34\,\mu\text{m}$ and $h = 2\,\mu\text{m}$, and panel conventions follow Supplementary S4.

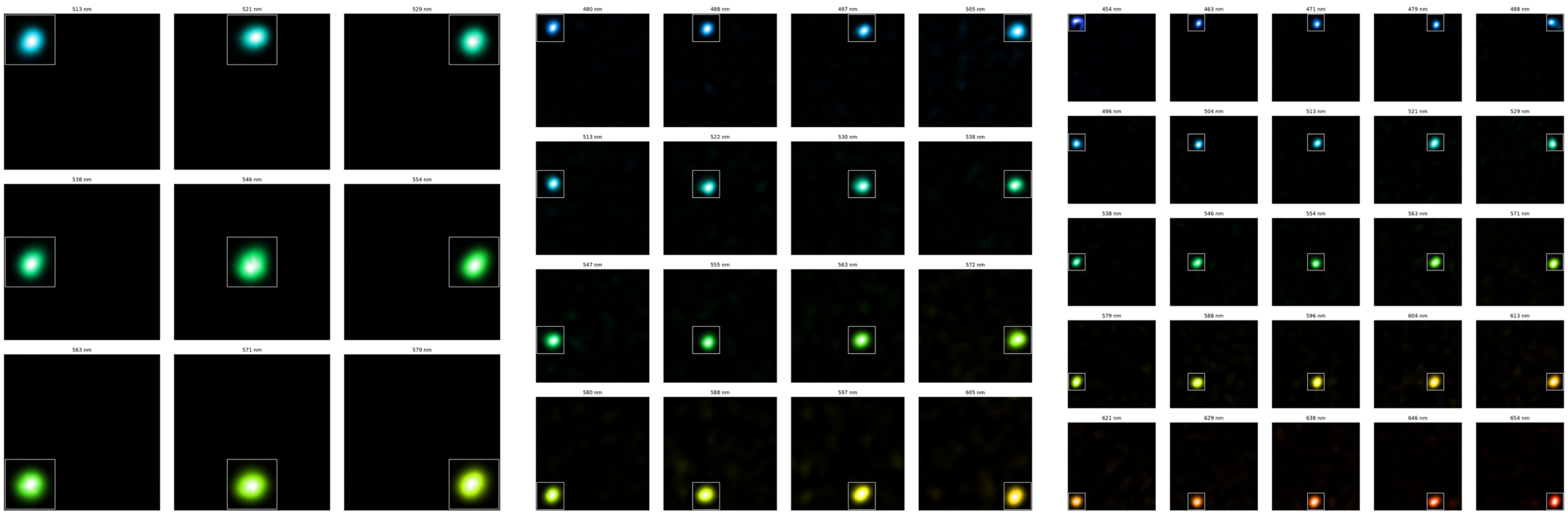

(a) 9 channels (b) 16 channels (c) 25 channels

Figure S13: Focal-plane S_z montages of the compressed-spacing control configurations (nominal $\Delta\lambda = 8\,\text{nm}$) for the (a) 9-, (b) 16-, and (c) 25-channel routers at $P = 0.34\,\mu\text{m}$. Each tile is labeled with its target wavelength. The white box in each map indicates the target sub-pixel region, and each map is normalized independently for visualization.

S9 Height Dependence and Worst-Channel Efficiency

This section examines how the average routing efficiency and the achieved worst-channel value $\rho_{0,\min} = \min_i T_{ii}(\lambda_i)$ depend on the device thickness h, complementing the fixed thickness $h = 2\,\mu\text{m}$ used in the main text. Figure S14 shows both quantities as functions of h at $P = 0.34\,\mu\text{m}$. The average routing efficiency increases steeply with thickness up to approximately $h = 2\,\mu\text{m}$ and varies only weakly, though not strictly monotonically, thereafter, consistent with the discussion of Fig. 2 in the main text. The worst-channel value $\rho_{0,\min}$ remains below the average for all channel counts.

Tables S6 and S7 provide the corresponding numerical values for the average routing efficiency and $\rho_{0,\min}$, respectively, including the additional sub-pixel sizes $P = 0.50\,\mu\text{m}$ and $0.60\,\mu\text{m}$. At $h = 2\,\mu\text{m}$ and $P = 0.34\,\mu\text{m}$, the difference between the average and worst-channel efficiencies increases monotonically with channel count, from 0.7 percentage points for $N = 9$ to 1.2, 3.8, and 6.7 percentage points for $N = 16$, 25, and 36, respectively, indicating that the worst-performing channel increasingly deviates from the average as more channels are routed simultaneously. The markers in Fig. 2 of the main text correspond, at each thickness, to the largest $\rho_{0,\min}$ over the available sub-pixel sizes in Table S7.

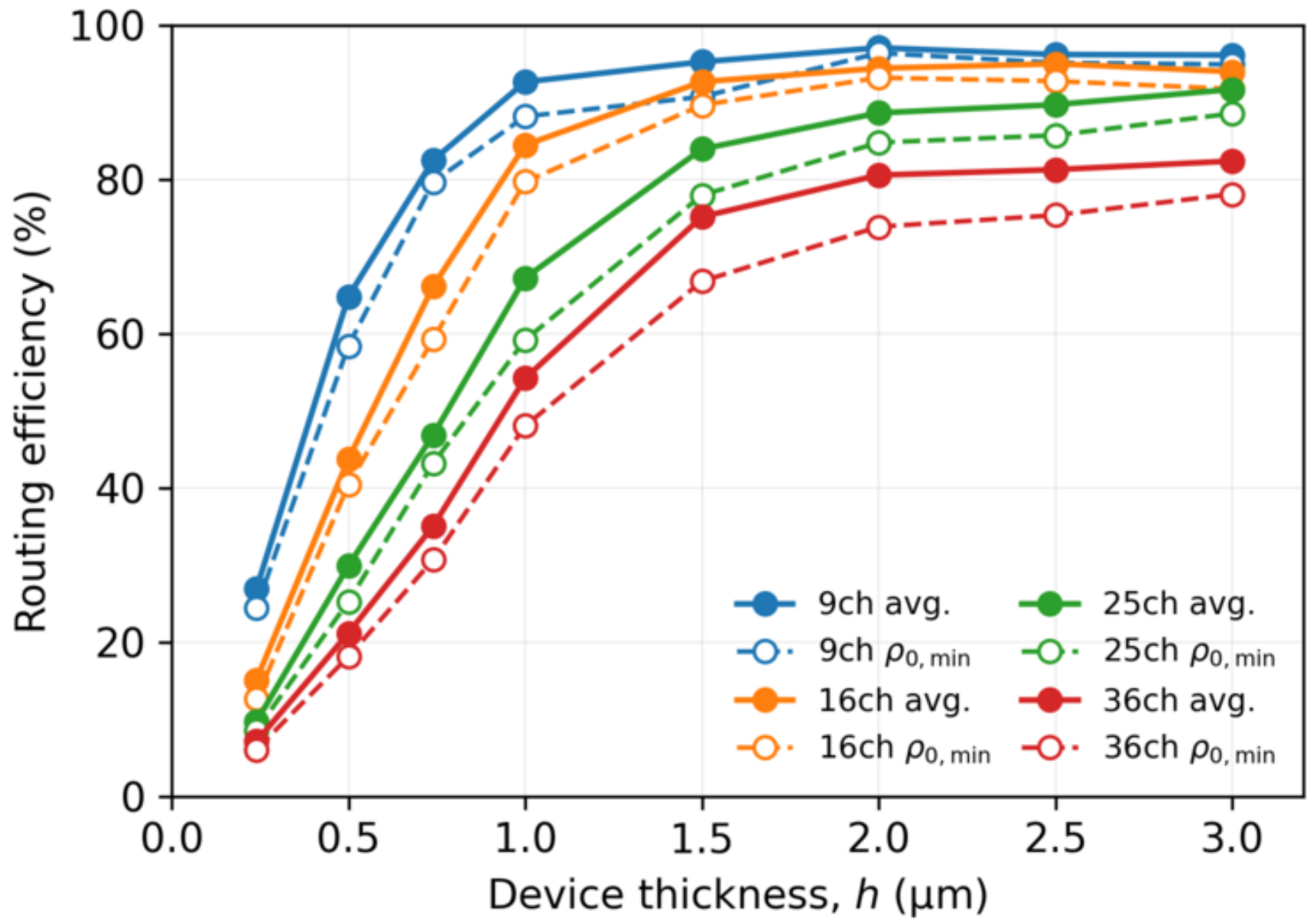

Figure S14: Average routing efficiency (solid lines, filled markers) and achieved worst-channel value $\rho_{0,\mathrm{min}}$ (dashed lines, open markers) as functions of device thickness h at $P = 0.34\,\mu\mathrm{m}$.

Table S6: Average routing efficiency (%) as a function of device thickness h for each channel count N and sub-pixel size P.

P	h (μm)	$N = 9$	$N = 16$	$N = 25$	$N = 36$
0.34 μm	0.24	26.91	15.09	9.69	7.12
	0.50	64.77	43.70	29.86	21.14
	0.74	82.46	66.12	46.86	35.03
	1.00	92.61	84.42	67.19	54.19
	1.50	95.21	92.63	83.90	75.12
	2.00	97.03	94.37	88.59	80.52
	2.50	96.16	94.96	89.66	81.24
	3.00	96.08	93.94	91.64	82.34
0.50 μm	2.00	96.10	94.02	89.31	82.29
	2.50	95.54	95.83	92.32	85.95
	3.00	95.05	95.07	91.46	86.35
0.60 μm	2.00	96.87	94.57	89.45	82.25
	2.50	96.60	94.01	92.41	85.99
	3.00	95.80	93.63	91.65	85.47

Table S7: Achieved worst-channel value $\rho_{0,\mathrm{min}}$ (%) as a function of device thickness h for each channel count N and sub-pixel size P.

P	h (μm)	$N = 9$	$N = 16$	$N = 25$	$N = 36$
0.34 μm	0.24	24.42	12.66	8.57	6.06
	0.50	58.40	40.37	25.22	18.10
	0.74	79.65	59.26	43.16	30.67
	1.00	88.13	79.67	59.16	48.01
	1.50	90.68	89.60	77.93	66.77
	2.00	96.35	93.14	84.75	73.83
	2.50	95.09	92.71	85.68	75.33
	3.00	94.88	91.70	88.51	78.06
0.50 μm	2.00	94.95	90.26	87.27	78.33
	2.50	94.84	94.98	90.75	81.83
	3.00	93.75	94.13	89.06	83.02
0.60 μm	2.00	95.76	92.19	87.58	77.14
	2.50	95.48	92.17	90.80	82.01
	3.00	93.87	91.34	89.52	81.00

S10 Transmission-Normalized Routing Efficiency

The routing efficiency reported in the main text is normalized to incident power. To assess how the transmitted power is distributed among the designated detector regions, we also define

$$\eta_i^{\text{tr}}(\lambda) = \frac{\Phi_i(\lambda)}{\Phi_{\text{out}}(\lambda)} = \frac{T_{ii}(\lambda)}{T_{\text{tot}}(\lambda)},$$

where Φ_i is the power delivered to detector region i, Φ_{out} is the total forward power crossing the output plane, and $T_{\text{tot}} = \Phi_{\text{out}}/\Phi_{\text{inc}}$. Here, Φ_{inc} denotes incident power. Whereas $T_{ii} = \Phi_i/\Phi_{\text{inc}}$ measures the fraction of incident power delivered to the target region, η_i^{tr} measures the fraction of transmitted power delivered to that region.

Figure S15 compares the two efficiencies for the 9-, 16-, 25-, and 36-channel routers at $P = 0.34\,\mu\text{m}$. Within each panel, the two curves for a given detector region use the same target power Φ_i and differ only in their normalization. At the designated wavelengths, the mean transmission-normalized efficiencies are 97.5%, 95.8%, 90.7%, and 83.4%, respectively. The decrease with channel count therefore persists when routing is evaluated as a fraction of transmitted power. The incident-normalized efficiency remains the performance metric used for the main channel-scaling comparisons.

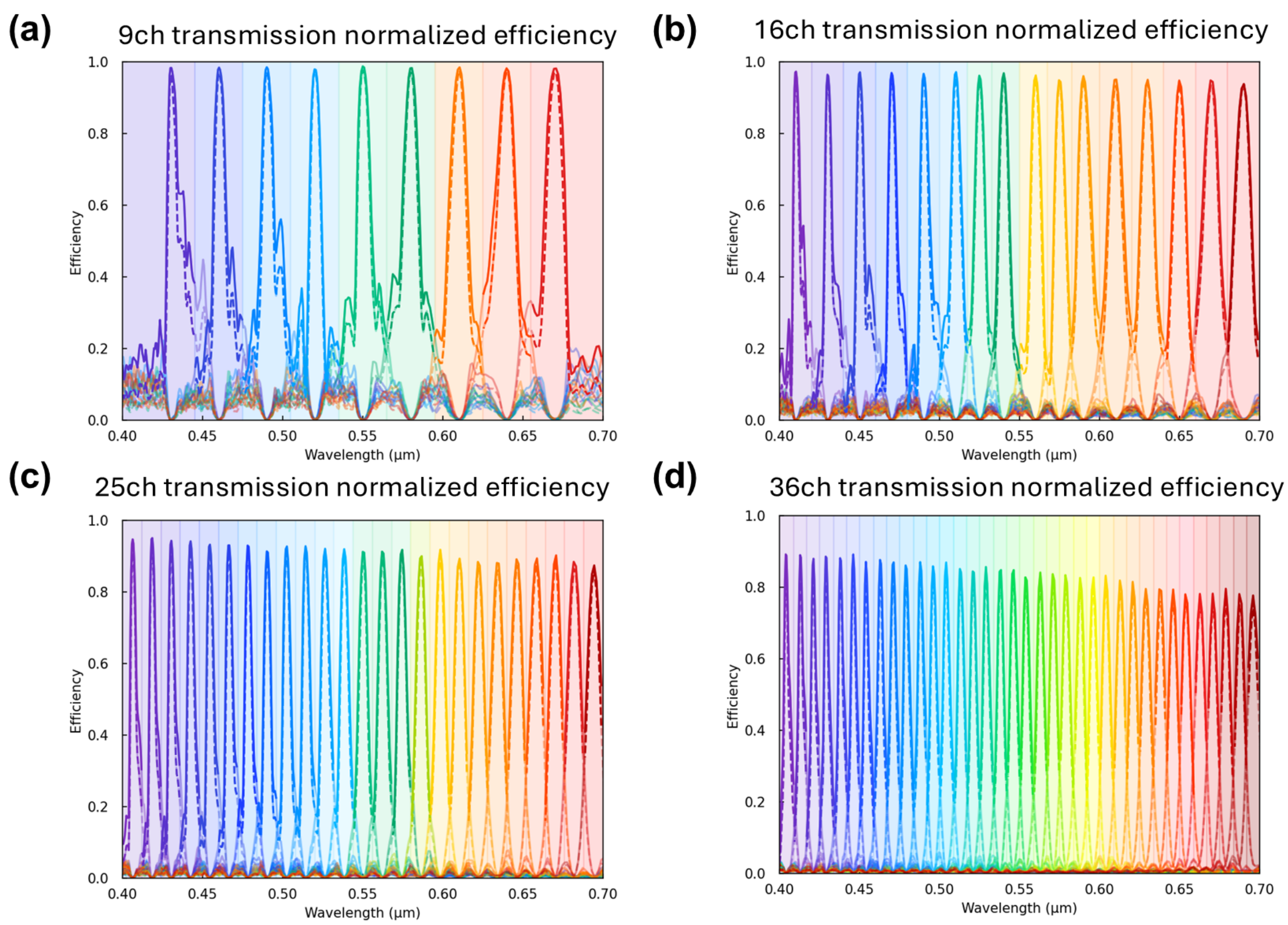

Figure S15: Transmission-normalized and incident-normalized routing-efficiency spectra for the (a) 9-, (b) 16-, (c) 25-, and (d) 36-channel routers at $P = 0.34\,\mu\text{m}$. Solid curves show the transmission-normalized efficiency $\eta_i^{\text{tr}} = \Phi_i/\Phi_{\text{out}}$, and dashed curves show the corresponding incident-normalized routing efficiency $T_{ii} = \Phi_i/\Phi_{\text{inc}}$. Each color denotes one designated detector region.

S11 Polarization Response of a Dual-Polarization-Optimized 36-Channel Router

To examine polarization dependence, we separately optimized a 36-channel router at $P = 0.34\,\mu\text{m}$ and $h = 2\,\mu\text{m}$ with both x- and y-polarized illumination included in the objective function, using the opti-

mization schedule in Table S1. This design is distinct from the 45°-polarization-optimized routers used in the main channel-scaling analysis.
Figure S16 shows the routing-efficiency spectra under 45° linearly polarized, x-polarized, and y-polarized illumination at normal incidence. The mean routing efficiencies at the 36 target wavelengths are 71.1%, 71.4%, and 70.7%, respectively, and the corresponding worst-channel efficiencies are 62.9%, 63.5%, and 63.1%. The similar responses indicate weak polarization dependence for this design under the tested conditions. Its 71.1% mean efficiency under 45° illumination is 9.4 percentage points lower than the 80.5% obtained for the 45°-polarization-optimized 36-channel router at the same sub-pixel size and thickness (Table S6). This difference is consistent with the additional routing constraints introduced by optimizing both polarizations, but it does not set a general limit on the efficiency cost of polarization robustness.

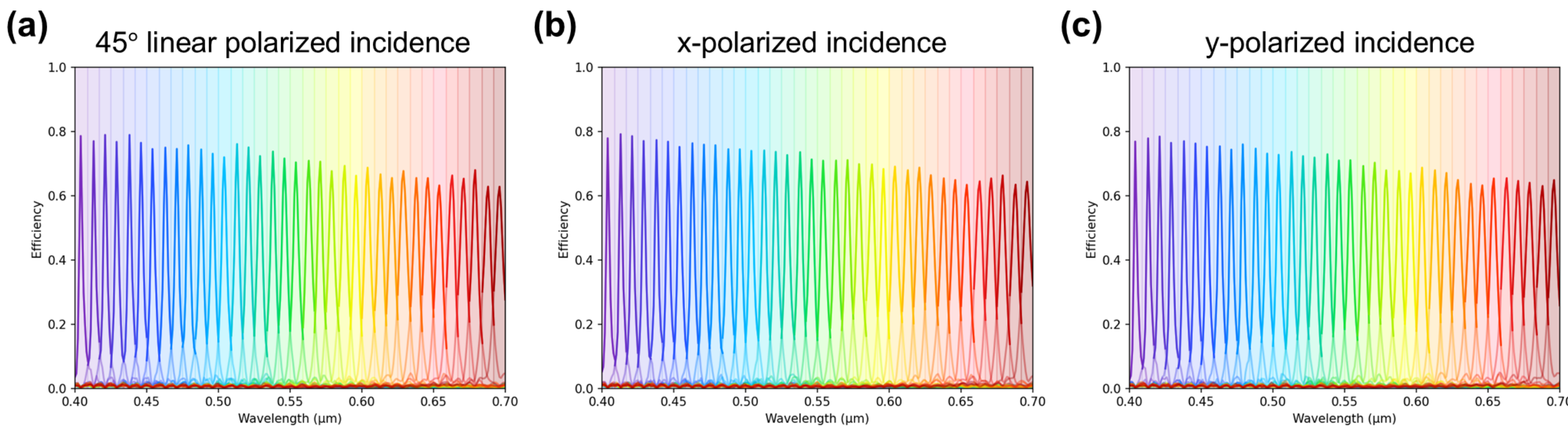

Figure S16: Polarization-resolved routing-efficiency spectra of a 36-channel router optimized for both x- and y-polarized illumination, with $P = 0.34\,\mu\text{m}$ and $h = 2\,\mu\text{m}$. The same structure is evaluated at normal incidence under (a) 45° linearly polarized, (b) x-polarized, and (c) y-polarized illumination. Each colored curve shows the fraction of incident power routed to a designated target sub-pixel as a function of wavelength.

S12 Visible-Band Routing with Refractive-Index Dispersion

To examine visible-band routing with refractive-index dispersion, we separately optimized a 36-channel router at $P = 0.34\,\mu\text{m}$ and $h = 2\,\mu\text{m}$. The 36 designated wavelengths span 404–696 nm. For TiO_2, we used DeVore's ordinary-ray Sellmeier relation for rutile [1],

$$n^2_{\mathrm{TiO_2}}(\lambda) = 5.913 + \frac{0.2441}{\lambda^2 - 0.0803}, \qquad \lambda \text{ in } \mu\text{m}.$$

The reported wavelength range of this relation is 430–1530 nm. Its use for the 404–430 nm channels therefore involves extrapolation. For SiO_2, we fitted a single-pole Lorentz model to the optical-constant data for glass compiled by Palik [2]. Panels (a) and (b) of Fig. S17 show the refractive-index curves used in the optimization. Both materials were represented by scalar real refractive indices. Material absorption and rutile birefringence were not included in this calculation.
Figure S17(c) shows the routing-efficiency spectra of the separately optimized router under normally incident 45° linearly polarized illumination. At the 36 designated wavelengths, the mean routing efficiency is 81.0%, and the worst-channel efficiency is 74.8%. The mean is close to the 80.5% obtained for the constant-index 36-channel router at the same sub-pixel size and thickness. The two structures were optimized separately using different material models, so this comparison does not quantify how the constant-index design responds to a change in material properties.

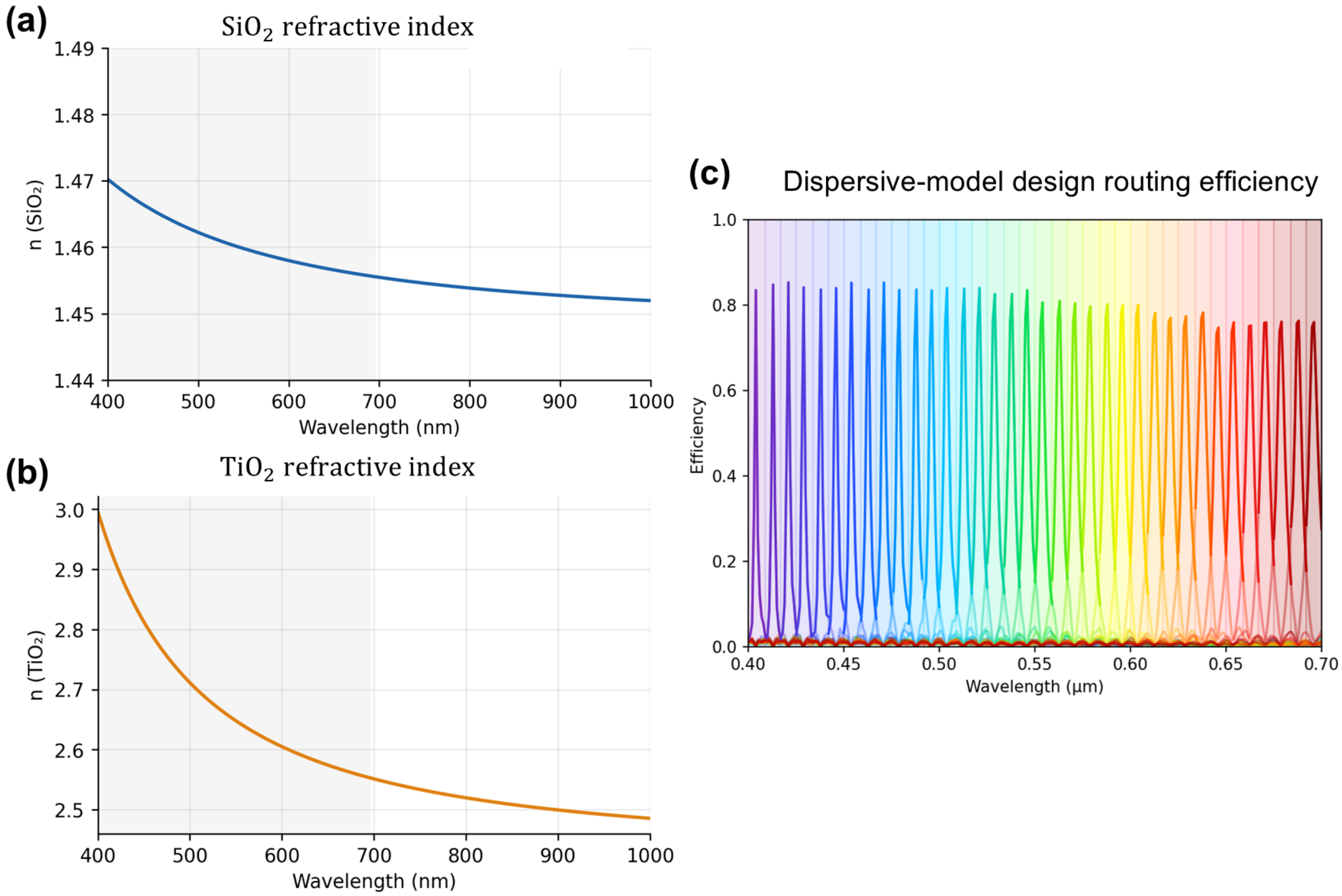

Figure S17: Visible-band routing using wavelength-dependent real refractive indices. (a,b) Indices used for (a) SiO_2 and (b) TiO_2, plotted over 400–1000 nm. (c) Routing-efficiency spectra of the separately optimized 36-channel router over 404–696 nm. Each colored curve shows the fraction of incident power delivered to a designated target sub-pixel as a function of wavelength.

S13 Extension to the Visible–Near-Infrared Band

This section presents a preliminary extension of the 36-channel router to the combined visible and near-infrared (VIS–NIR) band. The 36 target wavelengths are equally spaced between 404 nm and 996 nm (channel spacing of approximately 16.9 nm) and are assigned to the 6 × 6 sub-pixel array in row-major order from shorter to longer wavelengths, following the same convention as Table S2. The router uses a sub-pixel size of $P = 0.60\,\mu\text{m}$ and a device thickness of $h = 2\,\mu\text{m}$. All other design conditions are the same as in the visible-band 36-channel routers. For this preliminary VIS–NIR extension, the same wavelength-independent material indices as in Supplementary Sec. S1 were used.
To examine material dispersion, we separately optimized another 36-channel router with the same target wavelengths, sub-pixel size, and thickness using the wavelength-dependent, real-valued refractive-index models described in Supplementary Sec. S12. Material absorption was not included in either model. Figures S18(a,c) show the focal-plane S_z montage and routing-efficiency spectra of the constant-index design, and Figs. S18(b,d) show those of the dispersive-model design. The constant-index VIS–NIR router achieves an average routing efficiency of 79.9%, and the achieved worst-channel value is $\rho_{0,\min} = 72.2\%$, occurring at the longest-wavelength channel (996 nm). The per-channel efficiency decreases gradually toward longer wavelengths, from approximately 86% near 455 nm to approximately 72% near 996 nm.
The dispersive-model design achieves a mean routing efficiency of 77.8% and a worst-channel efficiency of 68.2% at approximately 962 nm. Its efficiency at the longest target wavelength, 996 nm, is 69.4%. Over the 12 channels spanning approximately 810–996 nm, the mean efficiencies of the constant-index and dispersive-model designs are 75.1% and 71.8%, respectively.
The separately optimized dispersive-model design therefore approaches the mean routing efficiency of the

constant-index design under the simulated conditions, although its longest-wavelength channels remain less efficient. The two structures were optimized separately, so this comparison does not quantify the effect of introducing dispersion into the original constant-index structure. The constant-index result, obtained with an idealized material model, remains preliminary.

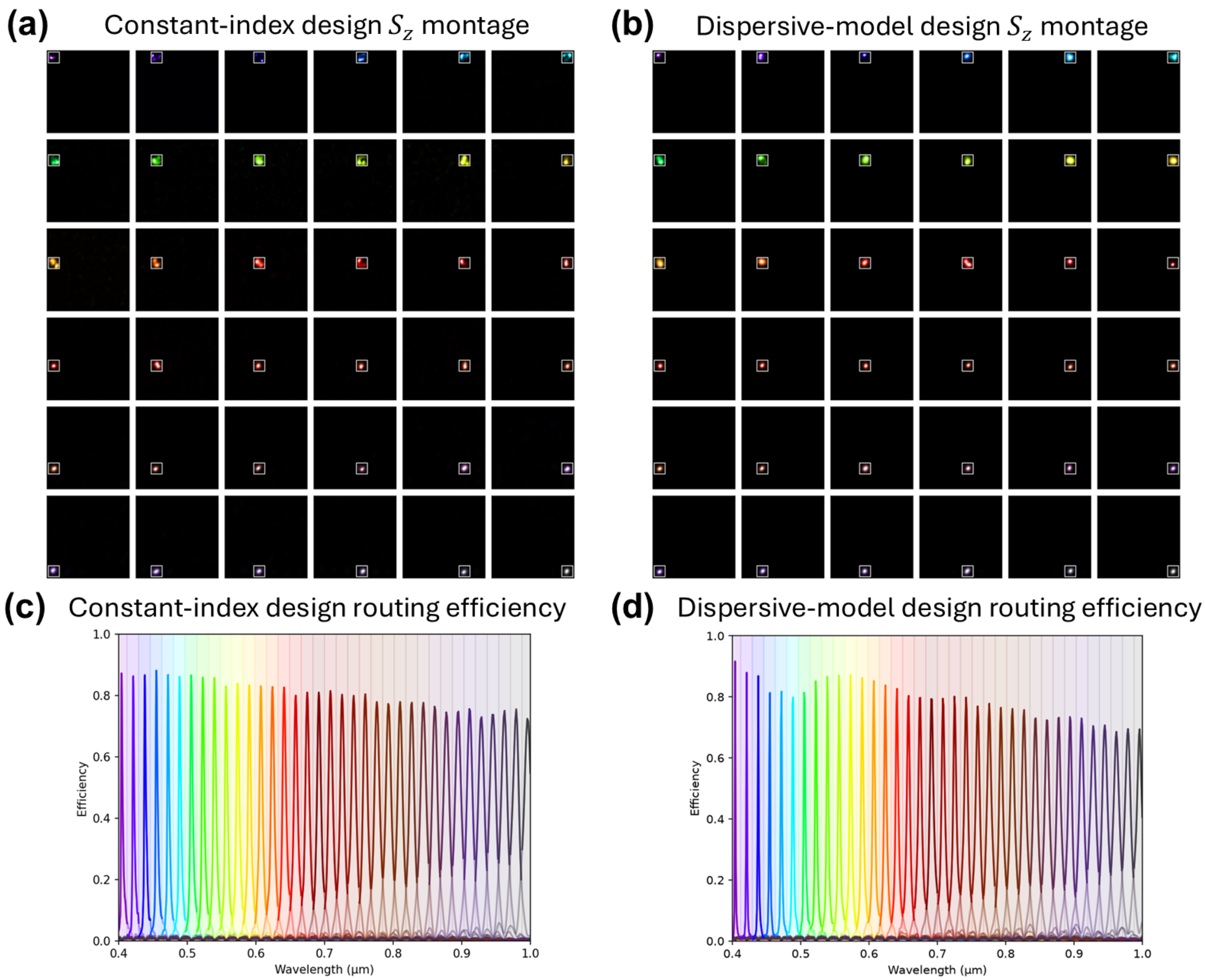

Figure S18: VIS–NIR 36-channel routers at $P = 0.60\,\mu m$ and $h = 2\,\mu m$. (a,b) Focal-plane S_z montages of the (a) constant-index and (b) dispersive-model designs. The maps are arranged in row-major order of increasing target wavelength. The white box in each map indicates the target sub-pixel region, and each map is normalized independently for visualization. (c,d) Routing-efficiency spectra of the (c) constant-index and (d) dispersive-model designs. The two designs were optimized separately. The routing efficiency of each channel is evaluated at its target wavelength.

S14 Fabrication Feasibility: Minimum-Feature-Size Constraint

This section examines the effect of the minimum feature size (MFS) imposed during optimization on the routing performance. The 36-channel router was re-optimized with minimum feature sizes of 20 nm, 60 nm, and 100 nm, enforced by the conic filter in the optimization flow of Supplementary S1. The 20 nm case corresponds to the baseline design. All other design conditions are kept the same as in the visible-band 36-channel baseline, including the sub-pixel size $P = 0.34\,\mu m$ and the device thickness $h = 2\,\mu m$. Figure S19 shows representative slices of the optimized design region and the corresponding routing-efficiency spectra for each minimum feature size. The average routing efficiency is 80.5% (MFS = 20 nm), 79.8% (MFS = 60 nm), and 62.8% (MFS = 100 nm), with achieved worst-channel values of $\rho_{0,\min} = 73.8\%$,

74.6%, and 56.8%, respectively. The average routing efficiency remains nearly unchanged up to a minimum feature size of 60 nm and decreases substantially at 100 nm.

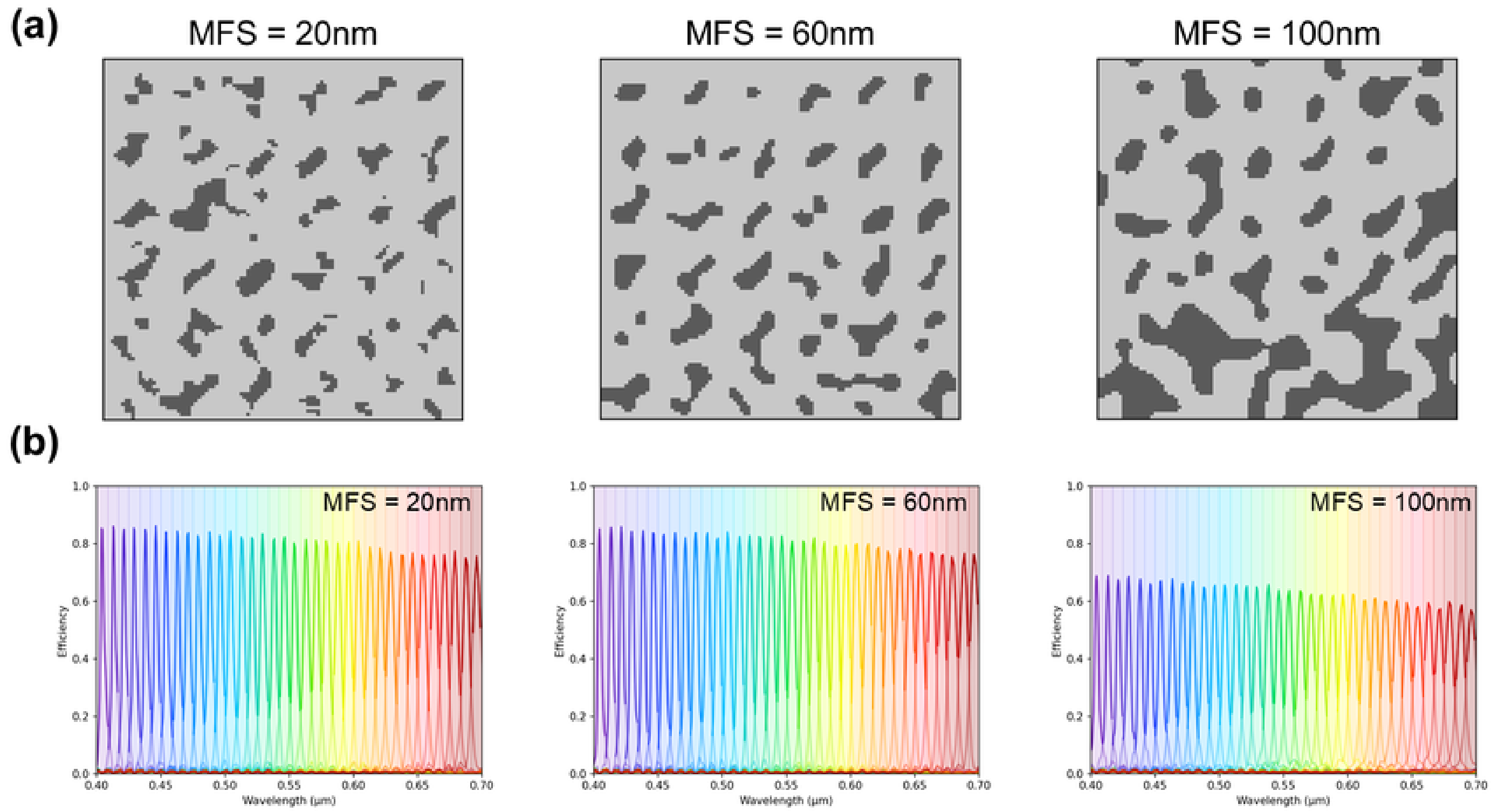

Figure S19: Minimum-feature-size dependence of the 36-channel router at $P = 0.34\,\mu m$ and $h = 2\,\mu m$. (a) Representative slices of the design region optimized with minimum feature sizes of 20 nm, 60 nm, and 100 nm. The light gray corresponds to SiO_2 and the dark gray to TiO_2. (b) Routing-efficiency spectra of the corresponding designs. The routing efficiency of each channel is evaluated at its target wavelength.

S15 Sensitivity to Uniform Interface Perturbations

We examined the sensitivity of the 36-channel router at $P = 0.34\,\mu m$ and $h = 2\,\mu m$ to uniform perturbations of its lateral TiO_2/SiO_2 interfaces. Erosion and dilation have been used to assess fabrication sensitivity in inverse-designed photonic devices [3, 4].

We assigned the arithmetic mean of the TiO_2 and SiO_2 permittivities to a band of width $d = 1$, 2, or 5 nm adjacent to each lateral interface [Fig. S20(a)]. Bands on the TiO_2 and SiO_2 sides define erosion-like and dilation-like perturbations, respectively. The bands are narrower than the approximately 20 nm design voxels and were therefore defined on an auxiliary xy grid with approximately 1 nm spacing. The permittivity was then averaged over each 20×20 block of the auxiliary grid to obtain one value per design voxel. The original interface positions and layer thicknesses were retained. For a locally planar interface, the change in spatially averaged permittivity is equivalent to moving a binary boundary by $d/2$. All structures, including the unperturbed reference, were simulated under identical conditions without reoptimization.

Figures S20(b,c) show the routing efficiency of each channel at its spectral peak as a function of the peak wavelength. For $d = 1$ nm, the mean peak efficiencies of the erosion-like and dilation-like cases are 80.3% and 79.8%, while the mean peak offsets from the target wavelengths are -2.5 and $+2.7$ nm, respectively. At $d = 2$ nm, the corresponding efficiencies are 78.9% and 77.5%, with offsets of -4.9 and $+5.4$ nm. At $d = 5$ nm, the efficiencies decrease to 70.8% and 67.1%, and the offsets reach -11.9 and $+13.0$ nm. These offsets exceed the approximately 8 nm spacing between adjacent target wavelengths.

For comparison, bands centered on the original interfaces, which approximately preserve the spatially averaged permittivity, produced mean peak offsets of only $+0.1$ and $+0.9$ nm for widths of 2 and 4 nm,

respectively. This comparison indicates that the larger offsets in the erosion-like and dilation-like cases arise mainly from the change in spatially averaged permittivity. The channel peak wavelengths are thus sensitive to uniform interface perturbations even when the mean peak efficiency remains relatively high. The reported efficiencies are evaluated at the shifted peaks and therefore do not represent routing performance at the original target wavelengths.

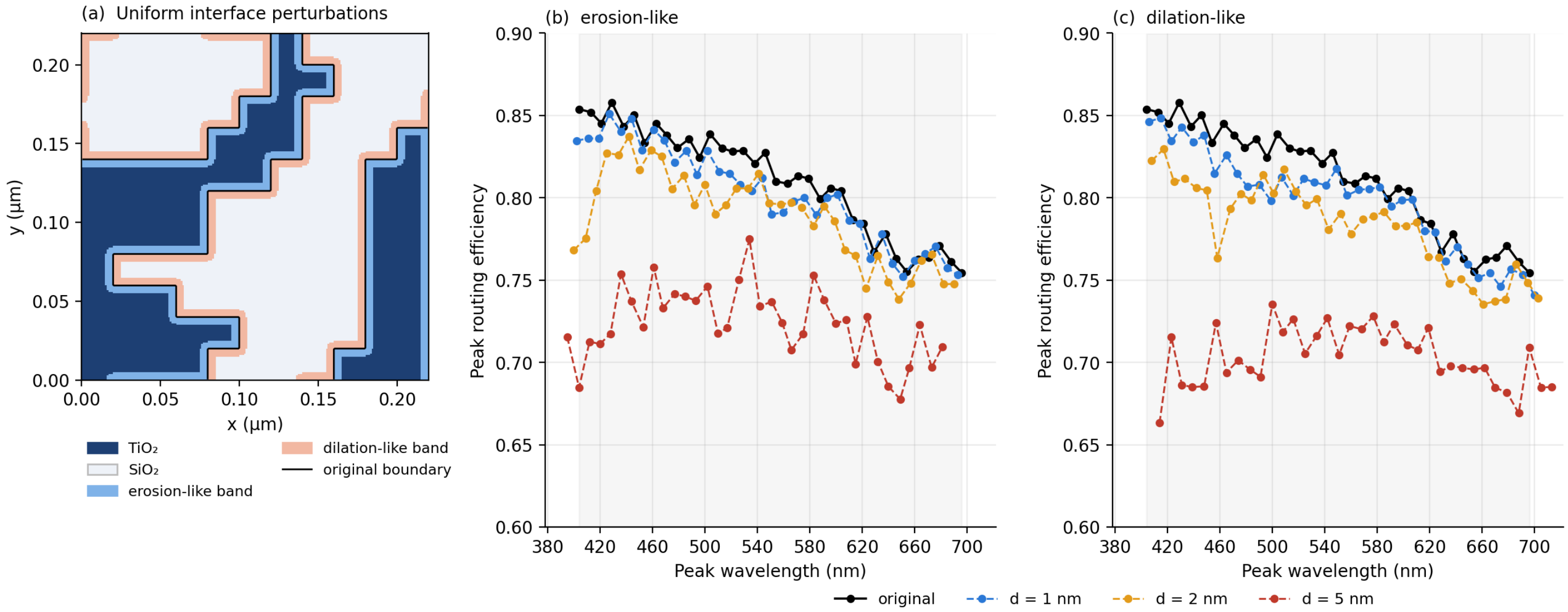

Figure S20: Response of the 36-channel router to uniform interface perturbations. (a) Magnified cross-section of a representative design region showing the erosion-like band on the TiO_2 side and the dilation-like band on the SiO_2 side for $d = 5$ nm, together with the original interface (black line). Each band has the arithmetic mean of the TiO_2 and SiO_2 permittivities. The two perturbations are shown together for comparison but were evaluated separately. (b,c) Peak routing efficiency versus peak wavelength for (b) erosion-like and (c) dilation-like perturbations. Black curves show the unperturbed structure, and colored curves show $d = 1$, 2, and 5 nm. Efficiencies are normalized to incident power.

References

[1] J. R. DeVore, Refractive indices of rutile and sphalerite, *Journal of the Optical Society of America* **1951**, *41*, 6 416–419.

[2] E. D. Palik, *Handbook of Optical Constants of Solids: Volume 1*, volume 1, Elsevier, **2012**.

[3] H. Li, N. Pyvovar, Z. Dai, O. D. Miller, Multimode grating couplers via foundry-compliant inverse design, *arXiv preprint arXiv:2604.28058* **2026**.

[4] H. Chung, F. Zhang, H. Li, O. D. Miller, H. I. Smith, Inverse design of high-NA metalens for maskless lithography, *Nanophotonics* **2023**, *12*, 13 2371–2381.
